# Genome-wide identification of meiotic recombination hot spots detected by SLAF in peanut (*Arachis hypogaea* L.)

**DOI:** 10.1038/s41598-020-70354-x

**Published:** 2020-08-14

**Authors:** Xiaohua Wang, Ping Xu, Yan Ren, Liang Yin, Shuangling Li, Yan Wang, Yanmao Shi, Hui Li, Xue Cao, Xiaoyuan Chi, Tianyi Yu, Manish K. Pandey, Rajeev K. Varshney, Mei Yuan

**Affiliations:** 1 College of Agriculture and Forestry Science, Linyi University, Middle of Shuangling Road, Lanshan District, Linyi, 276000 China; 2 Key Laboratory of Peanut Biology and Genetic Improvement, Ministry of Agriculture, Shandong Peanut Research Institute, No.126, Wannianquan Road, Licang District, Qingdao, 266100 China; 3 International Crops Research Institute for Semi-Arid Tropics (ICRISAT), Greater Hyderabad, Patancheru, 502 324 India

**Keywords:** Agricultural genetics, Polyploidy in plants

## Abstract

Recombination hot spots (RHP), caused by meiosis, are considered to play crucial roles in improvement and domestication of crop. Cultivated peanut is one of the most important rich-source of oil and protein crops. However, no direct scale of recombination events and RHP have been estimated for peanut. To examine the scale of recombination events and RHP in peanut, a RIL population with 200 lines and a natural population with 49 cultivars were evaluated. The precise integrated map comprises 4837 SLAF markers with genetic length of 2915.46 cM and density of 1.66 markers per cM in whole genome. An average of 30.0 crossover (2.06 cMMb^−1^) events was detected per RIL plant. The crossover events (CE) showed uneven distribution among B sub-genome (2.32) and A sub-genome (1.85). There were 4.34% and 7.86% of the genome contained large numbers of CE (> 50 cMMb^−1^) along chromosomes in F_6_ and natural population, respectively. High density of CE regions called RHP, showed negative relationship to marker haplotypes conservative region but positive to heatmap of recombination. The genes located within the RHP regions by GO categories showed the responding of environmental stimuli, which suggested that recombination plays a crucial role in peanut adaptation to changing environments

## Introduction

Peanut, also known as groundnut (*Arachis hypogaea* L.; AABB, 2n = 4x = 40), is a major source of edible oils worldwide, of which the annual production is about ~ 46 million tons (FAOSTAT 2015, https://faostat3.fao.org/home/). As a domesticated allotetraploid, the A subgenome and B subgenome of peanut are from the diploid species *A. duranensis* and *A. ipaensis* respectively, and it arised from natural hybridization of the two diploid species over 9400 years ago^1^. Cultivated peanut clusters the beneficial traits from the elites of germplasm. Limited by allotetraploidy and large genome (~ 2.7 Gb), it has been proven difficult to identify the required markers and to assemble a genome sequence in cultivated peanut (*A. hypogaea*)^1^.

In previous studies, quantitative trait loci (QTL) mapping has been a useful tool for dissecting the genetic architecture of complex traits in a great number of species. Many of those have focused on variation in important economic traits, such as oil content a polygenic, quantitative trait resulting from interactions between the environment and multiple genes^2,3^. For instance, a mass of QTLs that associated with important agronomic traits of *Arachis hypogaea* have been identified based on bi-parental linkage mapping. Nearly, 31 epistatic QTLs associated with five component traits of bruchid resistance throughout the total developmental period were screened in 2 years^4^. A total of 54 and 23 QTLs were identified for spotted wilt virus and leaf spot of tomato in the F_2_ and F_5_ peanut populations, respectively^5^. A total of 6 and 9 QTLs associated with oil content of peanut were identified in two different recombinant inbred line (RIL) populations respectively^6^. Recent improvements in sequencing technologies have reduced the cost of genotyping large numbers of accessions and increased the feasibility of performing pre-breeding at genome level as an alternative to QTL mapping or genome-wide associated studies.

The frequencies of recombination during crossover of homologous chromosomes could be detected by molecular markers, based on which a genetic map is constructed. Meiotic recombination which evolved into homologous chromosomes and the two sister chromatids formed after chromosomal replication could create genetic variation in gametes and new allele combinations^7,8^. As a fundamental biological process, meiotic recombination generates a new genetic diversity through alleles shuffling via crossover (reciprocal exchange of large chromosomal segments) or gene conversion (non-reciprocal exchange of small chromosomal segments), which impacts the genetic evolution of organisms^9^. So, recombination events is of great significance for the genomic evolution, especially for the crop domestication and improvement. For a offspring population, the allele distribution and haplotype structure is largely determined by the rate and distribution of recombination events happened in the whole genome of a species. The recombination crossover rates between species were different by the detection, for example, 20–50 crossover events per meiosis in human genome^2,10,11^; an average of 90 crossover events per meiosis w in budding yeast genome^12^; 81 crossover events per meiosis honey bee genome^13^; 10–37 crossover events per meiosis in *Arabidopsis* genome^14^ and 28–33 crossover events per meiosis in rice^15,16^. There were some regions showed uneven distributions of crossovers in the genomes. Some regions is entirely devoid the crossovers (0 cMMb^−1^) (cold spot crossover regions) and others showed the concentration of a large number of crossovers within a few kilobases (> 50 cMMb^−1^) (hot spot crossover regions)^7,8,14,15,16^. Many studies have shown that the hot spot crossover regions may contribute largely to evolution by generating novel heritable variations due to adaptive mutation or recombination^16^.

With lots of studies in diploid species, recombination crossover events in polyploid species are still unknown. To understand the crossing over of peanut, a RIL population (with 200 F_6_ lines) and a small natural panel (with 49 peanut cultivars) were used in our study. In total, 605,631 accurate markers, which were developed by specific-locus amplified fragment sequencing (SLAF-seq) approach^17^, were adopted to detect the recombination activity in the whole genome of F_6_ genetic map. A total of 61,942 InDels detected using transcriptome were adopted to detect the recombination activity in the whole genome of natural population. Together, present research aims to address the following objectives: (1) to construct a genetic linkage map of high-density SNP; (2) to detect the recombination crossover events in genetic map and natural population; (3) to identify the vicinity of genes located within the recombination hot spot regions by gene ontology (GO) categories.

## Results

### Quality control of specific-locus amplified fragment (SLAF) sequencing

**Figure 1:**
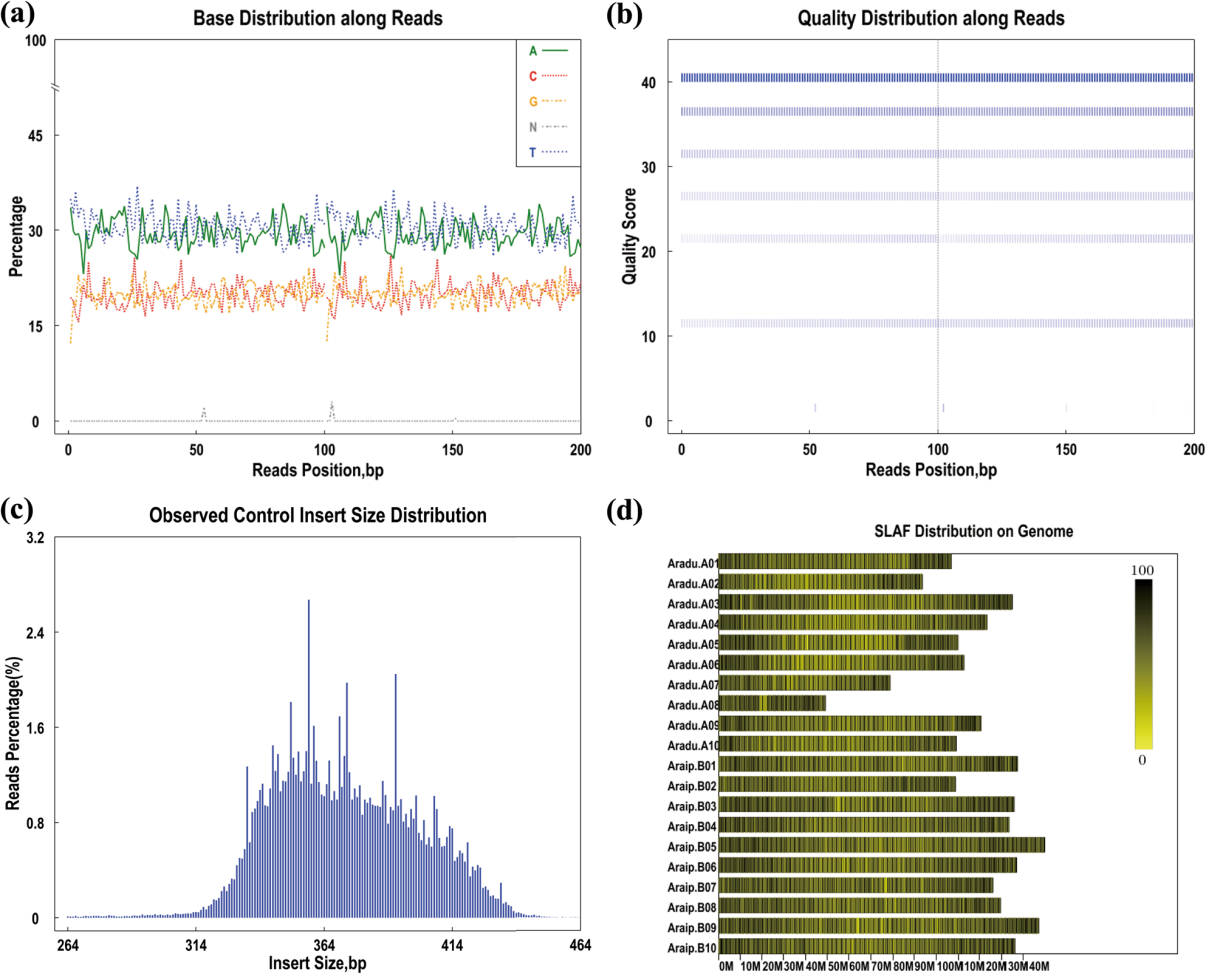
Specific-locus amplified fragment sequencing quality. (**a**) Base distribution along reads; (**b**) quality score of SLAF base calling; (**c**) control insert size of SLAF fragments distribution; (**d**) SLAF distribution on genome.

DNA fragments of 314–414 bp digested by *Rsa*l and *EcoR*V Enzymes were defined as SLAF tags. A total of 392,911 (with average of 19,646) SLAF tags located in a physical map of 2438.09 Mb were obtained by electronic digestion (Fig. 1d). The quality score of SLAF base calling were showed in Fig. 1b. The score of most base calling of SLAF showed equal to 40 (when Q ≥ 30, *P* value ≤ 0.001). To test whether the sequencing and libraries bring AT or GC base separation were good, the base distribution along reads were detected. The average of AT and CG equaled to 30 and 20, respectively (Fig. 1a), suggesting that the sequencing and libraries were of good quality. Because small insert size of SLAF fragments can lead to spurious mapping, the SLAF fragments with size from 264 to 464 bp (with average of 364 bp) were selected for preparation of libraries (Fig. 1c).

**Figure 2:**
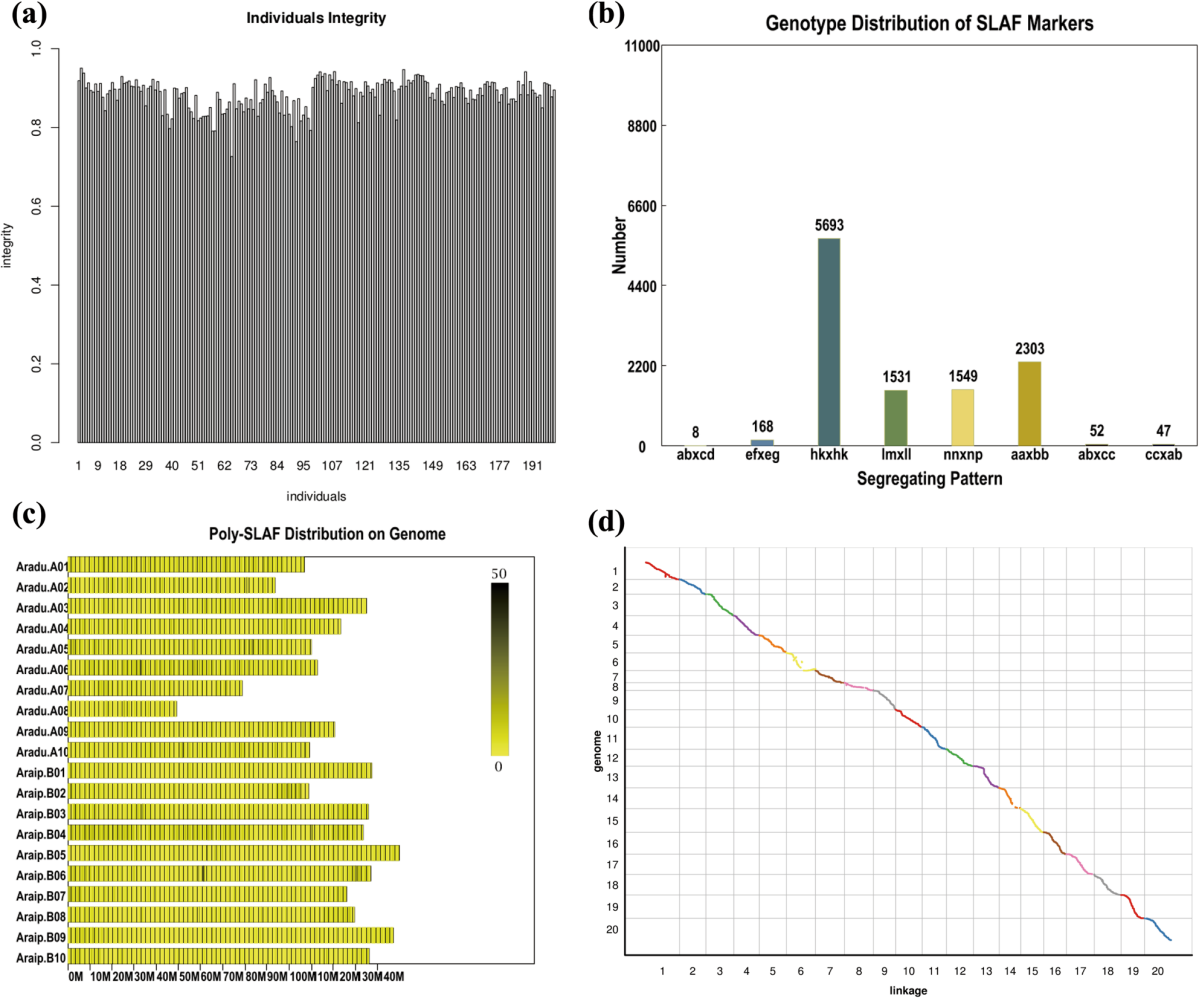
SLAF-markers selection and the construction of genetic linkage maps of RIL (F_6_) population. (**a**) The integrity of individuals in F_6_ population; (**b**) genotype distribution of SLAF markers; (**c**) polymorphic SLAF markers distribution on the physical map of peanut; (**d**) co-linearity of genetic linkage map and physical map of RIL (F_6_) population.

A total of 605,631 SLAF markers were developed by the SLAF-seq. The average individuals’ integrity was nearly 0.85 (Fig. 2a), suggesting that the markers were developed successfully. A total of 21,133 showed polymorphisms between cultivars 950527 and Huayu22 (Fig. 2c). Limited with the high similarity between A and B sub-genomes, the hemi-SNP type (hk × hk; lm × ll; nn × np) were selected and used for linkage map construction (Fig. 2b).

### Construction of a high-density SLAF based genetic linkage map.

**Figure 3:**
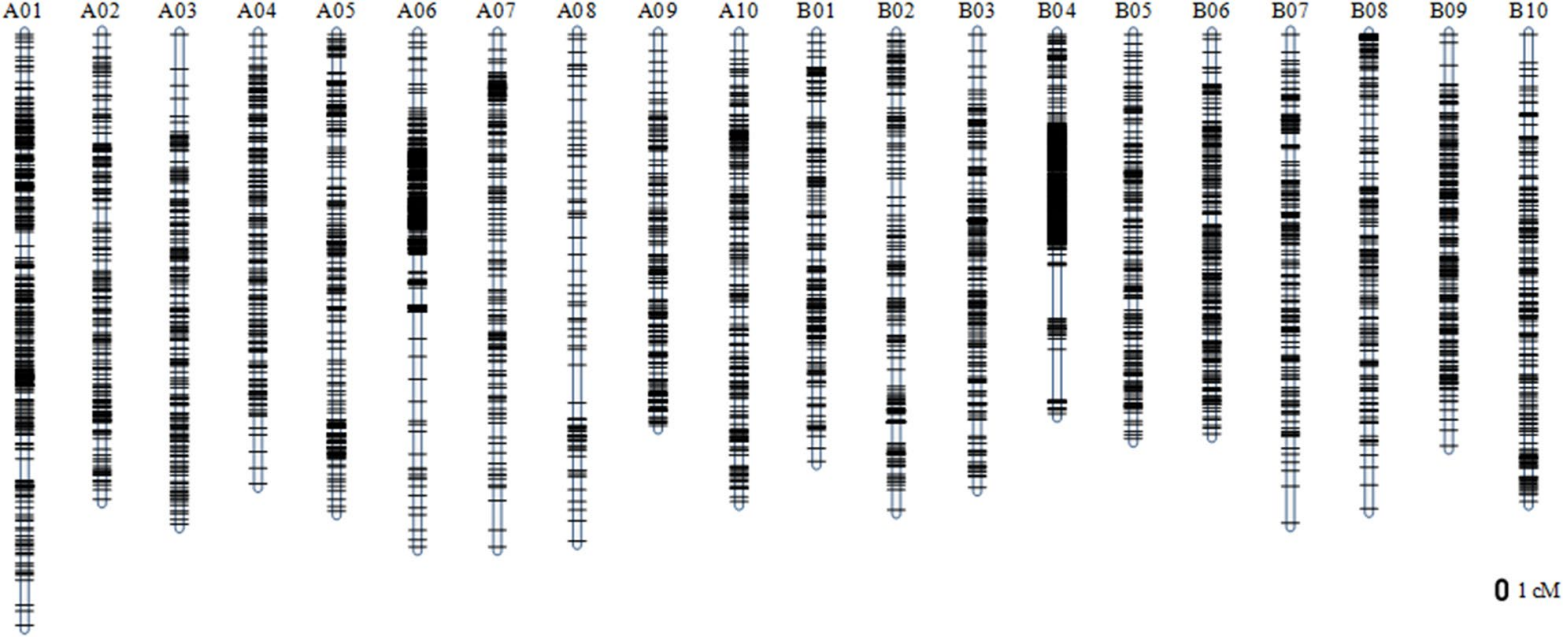
Genetic linkage map of RIL (F_6_) population. The black bars on each chromosome represent SLAF markers on the genetic linkage map.

A total of 11,076 poly-SLAF markers (normal and hemi-SNP) were developed by the SLAF-seq. Of these, 4837 SLAF markers with ≤ 5% missing data were successfully assigned to 20 linkage groups representing the A01-A10 and B01-B10 linkage groups of *A. hypogaea*, with total genetic distances of 1533.47 cM and 1381.99 cM for the A and B sub-genomes, respectively (Fig. 3; Table 1). The number of markers mapped on A and B sub-genome were 2383 and 2454, respectively (Table 1). The physical distance of A and B sub-genome were 1026.62 Mb and 1326.03 Mb. The marker number and density varied considerably with different chromosomes. The marker number ranged from 77 on chromosome A08 to 694 on chromosome B04 (with average of 241.85 per chromosome). The marker density ranged from 0.48 markers per cM on A08 to 5.80 markers per cM on B04 (with average density of 1.66 markers per cM). The quality of the linkage map could be detected by max gap and ratio of gap < 5 cM of each chromosome (Table 1). The collinear analysis of linkage map and physical map were showed in Fig. 2d.

**Table 1:** Number of molecular markers, genetic distance, marker density, max gap, ratio of gap < 5 cM, physical distance and recombination rates in each linkage group of the peanut F_6_ genetic linkage map.

Linkage group	Number of markers	Genetic distance (cM)	Marker density (markers/cM)	Max gap (cM)	Ratio of gap less than 5 cM (%)	Physical distance (Mb)	Recombination rates (cM/Mb)
A01	396	186.37	2.12	7.98	99.49	105.79	3.74
A02	179	146.82	1.22	5.05	99.44	91.89	1.95
A03	190	154.51	1.23	10.89	98.41	134.06	1.42
A04	180	141.79	1.27	5.36	98.88	122.25	1.47
A05	270	150.28	1.80	4.61	100.00	109.35	2.47
A06	505	161.58	3.13	8.65	98.21	110.91	4.55
A07	152	161.61	0.94	9.45	98.68	75.43	2.02
A08	77	159.90	0.48	11.95	92.11	48.00	1.60
A09	214	123.29	1.74	5.14	99.53	119.85	1.79
A10	220	147.32	1.49	5.08	99.54	109.09	2.02
B01	172	134.71	1.28	5.09	99.42	136.80	1.26
B02	174	149.98	1.16	6.46	97.69	105.81	1.64
B03	279	143.03	1.95	5.17	98.92	134.88	2.07
B04	694	119.66	5.80	16.84	99.71	129.59	5.36
B05	171	127.54	1.34	3.74	100.00	147.65	1.16
B06	215	126.04	1.71	4.82	100.00	136.43	1.58
B07	178	154.25	1.15	11.73	98.87	125.76	1.42
B08	185	149.64	1.24	7.44	98.91	128.40	1.44
B09	199	129.82	1.53	7.21	98.48	145.44	1.37
B10	187	147.32	1.27	8.88	99.46	135.29	1.38
A subgenome	2383	1533.47	1.55	11.95	98.43	1026.62	2.32
B subgenome	2454	1381.99	1.78	16.84	99.15	1326.02	1.85
Total	4837	2915.46	1.66	16.84	98.79	2352.64	2.06

### Identification of recombination events and recombination hot spots

**Figure 4:**
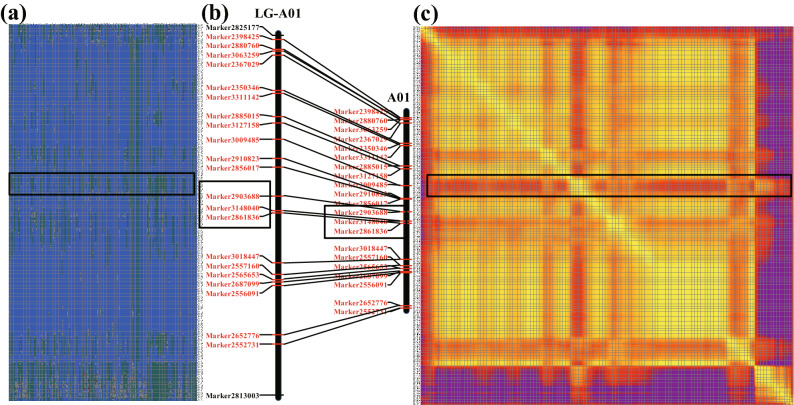
Recombination hot spots in the RIL (F_6_) population. (**a**) Haplotype maps of SLAF markers in RIL (F_6_) population; (**b**) co-linearity of recombination hot spots in genetic linkage map and physical map of RIL (F_6_) population; (**c**) heat maps of SLAF markers in RIL (F_6_) population.

The linear analysis was performed between the number of recombination events and the physical length of the chromosomes. The mean number of recombination events per chromosome pair was positively correlated with the physical length of the chromosome in the peanut F_6_ RIL population (Fig. 4; r = 0.99, *P* = 0.00062) and 49 peanut accessions (Table 2; r = 0.72, *P* = 0.00039), indicating that longer chromosomes have more recombination events. The recombination rate of each linkage group ranged from 1.16 cM per Mb on B05 chromosome to 5.36 cM per Mb on B04 (with an average of 2.06 cM per Mb) in the F_6_ RIL population (Table 1). The recombination rate of each chromosome ranged from 6.10 n per Mb on A04 to 15.71 n per Mb on A08 chromosome (with an average of 8.40 n per Mb) (Table 2).

**Table 2:** Number of recombination, star position, end position, physical distance and recombination rates in each chromosome of the peanut 49 accessions.

Chromosome	Number of recombination	End position (bp)	Start position (bp)	Distance (bp)	Distance (Mb)	Recombination rate (n/Mb)
A01	743	105,923,929	287,710	105,636,219	105.64	7.03
A02	666	92,619,538	32,207	92,587,331	92.59	7.19
A03	1154	133,100,052	57,469	133,042,583	133.04	8.67
A04	731	119,909,962	36,037	119,873,925	119.87	6.10
A05	932	108,244,824	9,936	108,234,888	108.23	8.61
A06	824	110,568,156	84,772	110,483,384	110.48	7.46
A07	575	77,818,488	106,281	77,712,207	77.71	7.40
A08	767	48,912,733	87,493	48,825,240	48.83	15.71
A09	782	118,990,609	13,322	118,977,287	118.98	6.57
A10	702	107,163,086	11,205	107,151,881	107.15	6.55
B01	1102	136,834,735	135,664	136,699,071	136.70	8.06
B02	977	108,513,667	169,195	108,344,472	108.34	9.02
B03	1718	135,528,322	75,576	135,452,746	135.45	12.68
B04	1094	133,156,247	52,357	133,103,890	133.10	8.22
B05	1313	149,413,871	104,928	149,308,943	149.31	8.79
B06	1248	136,646,814	75,883	136,570,931	136.57	9.14
B07	1082	125,895,165	69,248	125,825,917	125.83	8.60
B08	957	129,061,483	99,756	128,961,727	128.96	7.42
B09	1225	146,488,426	24,230	146,464,196	146.46	8.36
B10	1218	135,754,341	79,242	135,675,099	135.68	8.98
A subgenome	7876	1,023,251,377	726,432	1,022,524,945	1022.52	7.70
B subgenome	11,934	1,337,293,071	886,079	1,336,406,992	1336.41	8.93
Total	19,810	2,360,544,448	1,612,511	2,358,931,937	2358.93	8.40

**Figure 5:**
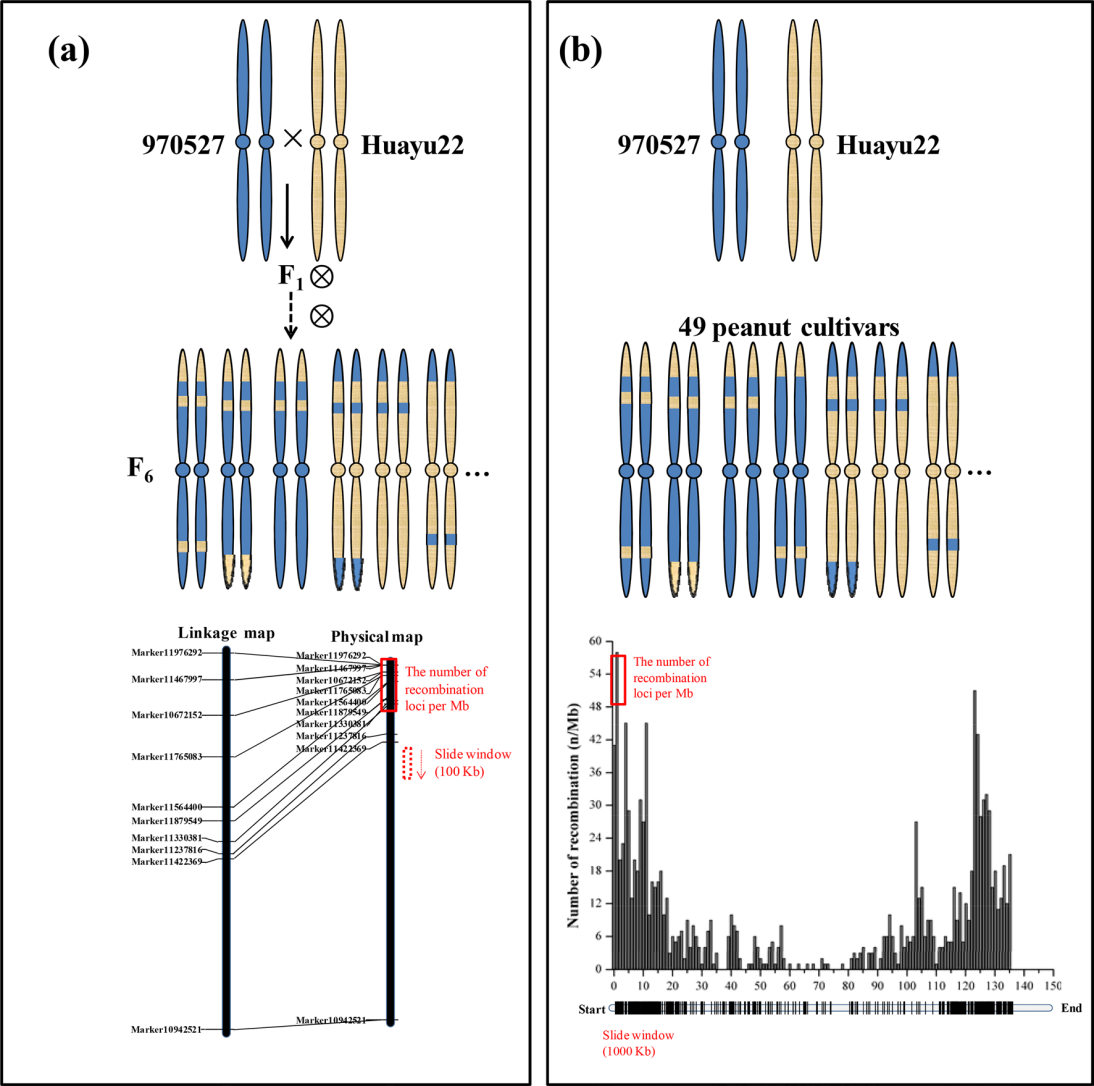
Genotyping and identification of crossover events in RIL (F_6_) population and 49 peanut accessions. (**a**) Identification of crossover events and recombination hot spots detection in RIL (F6) population; (**b**) identification of crossover events and recombination hot spots detection in 49 peanut accessions.

**Figure 6:**
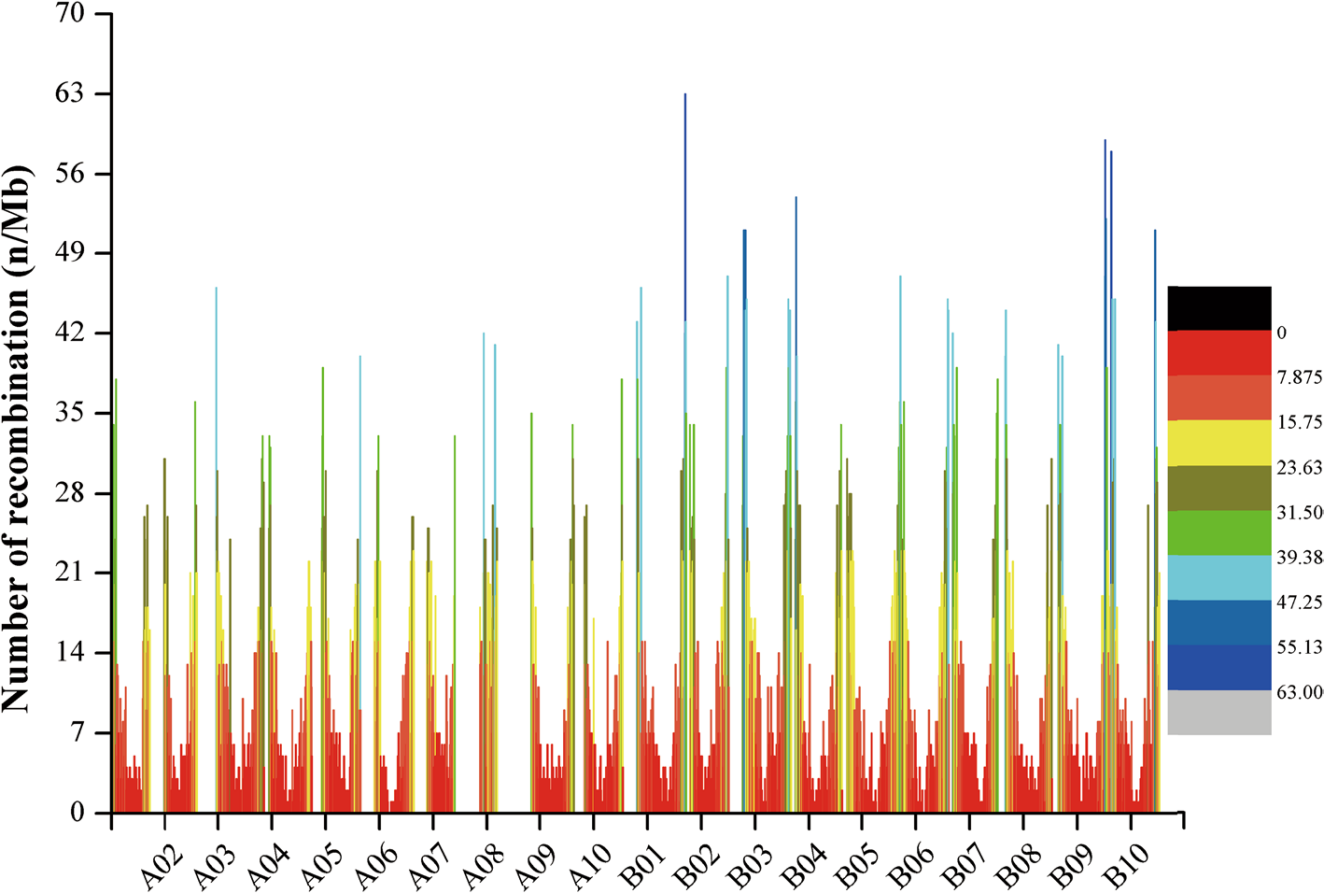
Recombination rates and hot spots of 49 peanut accessions. Different colors show the different recombination rates in the 49 accessions population; the recombination hot spots were defined as recombination > 25 n/Mb.

The SLAF marker haplotypes of each individual in the F_6_ linkage map was shown in Fig. 4a, and this suggests the recombination frequently regions mapped on genome. The relative recombination of each two markers was valued in the RIL (F_6_) linkage maps (Fig. 4c). Within linkage group, there were 4837 recombination crossover events detected (Fig. 5a). The number of recombination crossover events in the natural population was 2073 (Figs. 5b, 6, S1). The recombination frequency regions (recombination hot spots) on chromosomes are shown in Figs. 4 and S1. A total of 210 (4.34% of total recombination crossover events) of the loci were recombination hot spots in RIL population (Figure S1). Those recombination hot spots were located in 120 loci (2.35% of total recombination crossover events of total recombination crossover events) in A genome and 90 loci (1.86%) in B genome (Fig. 4). There were 163 (was 7.86% of whole recombination crossover events) regions which were recombination hot spots in the peanut natural population. The number of recombination hot spots was 49 (2.36% of all recombination crossover events regions) and 114 (5.50% of all recombination crossover events regions) located A and B genome (Figs. 6, S1), respectively.

### Functional characterization of genes with a high recombination rate

A total of 3865 genes were located in the interval (100 kb) of recombination hot spot loci. Nearly 841 types of functions could be classified by using peanut Gene Ontology annotation (https://geneontology.org/).

**Figure 7:**
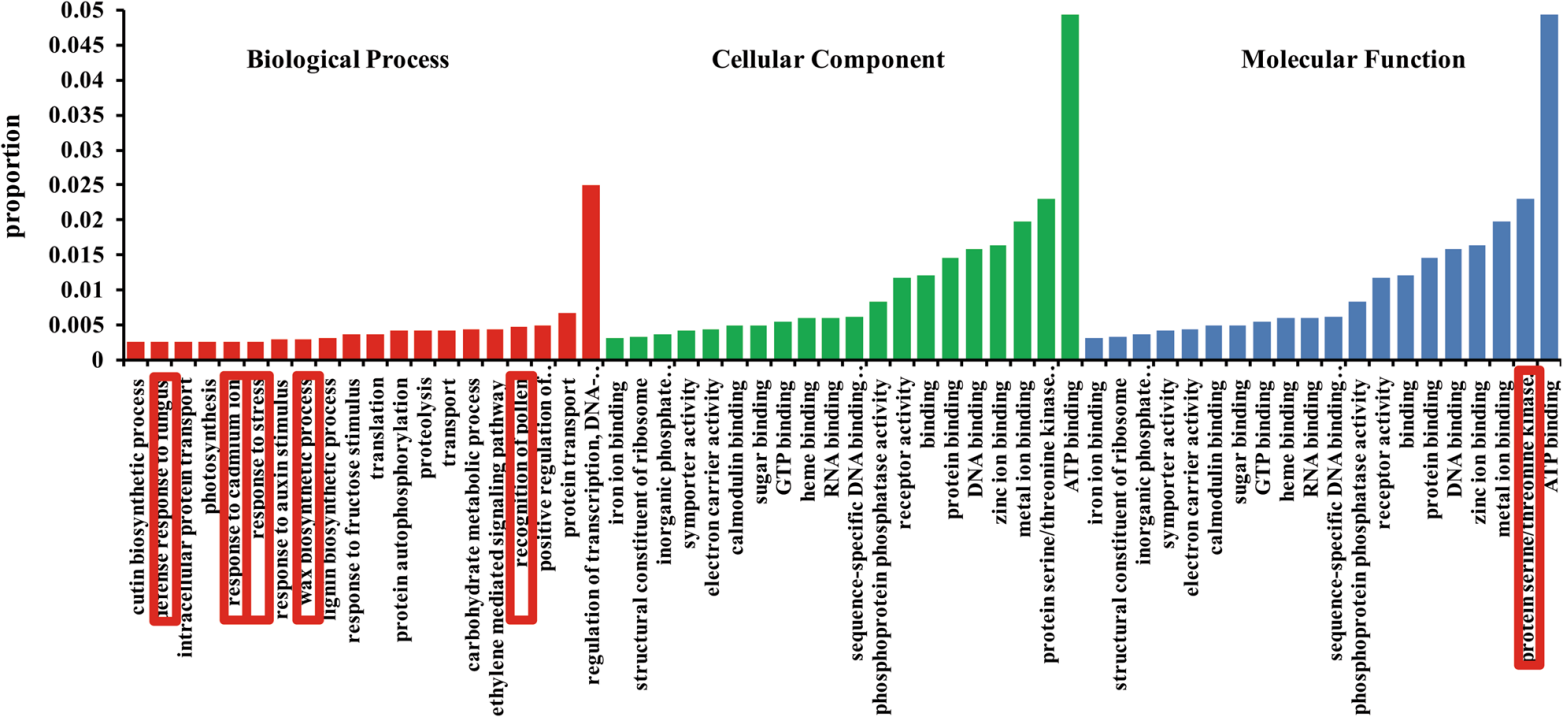
Functional categories of the genes with a high recombination rate.

The top 60 functions (54.30% of total genes function) were shown in Fig. 6. The genes could be divided into three functions including biological process (9.49% of total genes), cellular component (22.41% of total genes) and molecular function (22.41% of total genes) (Table S1). Most genes in cellular component and molecular function were involved into synthesis of ATP, DNA, RNA, and protein binding. Most of these genes in biological process have functions responding to the environmental stimulus (Fig. 7), for example, response to cadmium ion, stress, auxin stimulus, fructose stimulus, wax biosynthetic process, fungus, , etc., suggesting that the genes located in the region of high recombination rates on chromosome tend to be involved in the response to environmental stimuli and biotic stress . The frequent recombination in peanut RIL population and natural population may be beneficial to the adaptation of peanut in changing environments.

## Discussion

### High density SNP-based genetic linkage map construction

Cultivated peanut is an allopolyploid crop of which the genome structure iscomplex. A large number of homologous sequences exist between the peanut A- and B-subgenome, and simultaneously both the homologous and non-homologous exchanges are extensively observed between them^1^. This complexity leads to the difficulty of developing high-quality molecular markers used in association studies. Recent advances in sequencing technologies have reduced the cost of genotyping large numbers of accessions and increased the feasibility of constructing a high density, SNP-based genetic linkage map as an alternative to more traditional genetic mapping.

Genetic linkage maps could provide valuable references for studying genome structure and evolution, analyzing comparative genomes and localizing genes of interest^18^. In our study, a genetic linkage map of peanut with high density SNP markers was constructed (Fig. 3; Table 1). The number of markers mapped onto A- subgenome (2383 markers) was roughly same as those mapped onto B-subgenome (2454 markers) (Table 1). The physical and genetic distances between markers on the A genome were the same as on the B genome. The length of the genetic map in our studies was 2915.46 cM, which bigger than the lengths of 1935.4 cM^6^ and 1446.7cM^19^ published. The total number of markers mapped onto the genetic linkage map was 4837, which is larger number than 1267 bin marker^19^ and 418 markers^3^ mapped to genetic linkage maps previously. According to Zhou et al.^19^ and our results, the recombination distance in the A and B genome showed balance which is different from that observed in other polyploid species, such as *Brassica napus* (The recombination distance on A genome is greater than on C genome), indicating similar evolutionary history of *Arachis duranensis* and *Arachis ipaensis*^20,21^*.* Moreover, there appear to have been no further interspecific hybridization events during cultivated peanut breeding^1^.

### Recombination detected in peanut RIL and natural population

Meiotic recombination as an important, fundamental biological process can produce crossover and gene conversions, which greatly influences the genomic evolution, particularly the process of crop improvement or breeding. There were 24,185 crossover events per plant detected in the RIL population (Table 1), which is lower than that in rice^16^; that in budding yeast^12^; that in honey bee^13^ and that in *Arabidopsis*^15^. However, crossover events per plant species may not be suitable scales for detecting recombination crossover events across the genome. Because population crossover events depend on the population size, the density of recombination rate was a better scale for valuing genome crossover events. In our study, the average number of crossover events was 2.06 cM/Mb (Table 1), smaller than that in rice 4.53 cM/Mb^16^, suggesting a negative trend between genome size and recombination crossover events rates. Similar recombination crossover rates were observed in yeast and honey bee^14,15^. There was a weak correlation between genetic distance and recombination crossover rate in the RIL population (r = 0.1549, *P* = 1.08 × 10^–32^). There was also a weak correlation between genetic distance of each linkage group and recombination crossover rate (r = 0.1265, *P* = 1.35 × 10^–14^) too. The number of recombination showed a weak correlation between density of recombination crossover events rate and physical distance in 49 peanut cultivars (r = 0.3033, *P* = 7.71 × 10^–15^) (Table 2), suggesting that the recombination crossover rates are too complex to be controlled by one element. There was a strong correlation between recombination crossover events and physical distance in RIL and natural populations (Table 1).

Recombination hot spots were defined as the regions which showed high distributions of crossovers in the genomes^7,8^. In our studies, nearly 4.34% and 7.86% of recombination events on the genome were recombination hot spots (Figs. 4, 6), which are larger than those in rice (0.72% recombination hot spots of recombination crossover events)^16^. Compared with the RIL population, the natural population had higher recombination hot spot rates. The recombination hot spot rates on the A genome are larger than on the B genome in RIL population, suggesting more diversity within the A genome has been selected in the breeding process. However, the recombination hot spots rates were larger in the B genome than the A genome in the natural population, suggesting that there has been more balanced selection between the two genomes in nature.

### Higher recombination rates in the RIL and natural population and in the vicinity of environment-related genes

As an important polyploid edible oil crop, the recombination crossover events in the peanut genome appear to have played a crucial role in crop environment adaptation, breeding and improvements. Previous studies have shown that the genes in the hot spot crossover regions tended to be involved in responses to environmental stimuli^8,15^. In our work, a total of 3865 genes were located in the recombination hot spots regions. Most genes (accounting for 22.41% of total genes) were involved in cellular components and 22.41% of total genes in molecular function (Fig. 7; Table S1). Most genes in cellular components and molecular functions were involved in ATP, DNA, RNA, and protein binding, suggesting that the genes within hot spot regions have played an important role in maintaining normal regulation mechanisms of peanut. The genes involved in biological processes within recombination hot spot regions primarily tended to be involved in responses to environmental stimuli. This suggests that frequent recombination plays an important role in adaptive evolution in changing environment of peanut breeding or improvement (Fig. 7). Compared with the GO analysis in rice^16^, the GO analysis likely provides a pool of highly dynamic targets for selection, which is potentially a result of the elevated recombination rates in peanut.

## Experimental procedures

### Plant materials and DNA isolation

The RIL (F_6_) population including 200 peanut (*Arachis hypogaea* L.) lines was obtained from the crossing between female parent 950527 and male parent Huayu22. A total of 49 peanut accessions made up a natural population, which were collected from peanut breeding or improvement programs. All of the 249 genotypes were grown in a field screening nursery at Qingdao, China (120.41°E, 36.39°N) in May of 2017. Each accession was planted in a single-row with 12 individual plants within each row. The trial management followed standard breeding field protocols. Young leaves from one individual plant of each accession were collected and kept at − 80 °C freezer, and genomic DNA was isolated using CTAB method^22^.

### Genotyping of the RIL population

SNP genotyping of the association panel was performed using a SLAF-seq approach^17^. Construction of the peanut DNA libraries and Illumina sequencing of the plants were performed at Biomarker Technologies Corporation in Beijing, China. Through restriction enzymes HaeIII and Hpy166II (New England Biolabs, NEB, USA) that digest peanut genomic DNA into DNA fragments of 364–464 bp^23^, the sequencing libraries of 202 peanut accession were constructed. The physical position of the markers were identified by aligning the sequence of a 125 bp paired-end reads attached to each marker with the ‘pseudomolecules’ genome sequences of diploid peanut (*Arachis duranensis*-AA and *Arachis ipaensis*-BB, https://www.peanutbase.org) using local BLASTn (BLAST: Basic Local Alignment Search Tool, https://blast.ncbi.nlm.nih.gov/Blast.cgi). If the reads matched two or more locations in the reference genome of peanut, the markers were regarded as non-specific markers and discarded. Accurate markers were selected throught three steps. First, all candidate markers must be called in the mixed reads from parents and all the F_6_ samples using GATK (https://software.broadinstitute.org/gatk/). Second, all candidate markers must be called less than 20%. Third, some hemi-SNPs which showed polymorphism in sub-genomes were used for polymorphism markers in genetic linkage map in RIL population.

### Genotyping of the natural population

Peanut young seed of three plants of each of the 49 accessions were sampled 30–40 days (pegs stage) after flowering. Samples were cleaned and immediately placed in liquid nitrogen before being stored at − 80 °C^24^. The sequencing libraries of 147 RNA samples were generated using the Illumina RNA Library Prep Kit (NEB #E7760, San Diego, CA USA) and sequenced on an Illumina Hiseq 2000 platform with 100-bp paired-end reads. The physical position of the markers was identified by aligning the sequence of a 100 bp paired-end reads attached to each marker with the ‘pseudomolecules’ genome sequences of peanut using local BLASTn. The InDels markers with call frequencies > 0.8 and minor allele frequencies (MAF) > 0.05^25^ were selected for recombination crossover events analysis.

### Construction of genetic maps, heat maps and haplotype maps in RIL population

A representative SNP marker of a bin was selected to construct the genetic linkage map using the sofware packages of HighMap^13^. The map evaluation module provide heat maps and haplotype maps for intuitive displays of map quality. The grouping module uses the single-linkage clustering algorithm to cluster the markers into linkage groups, using a pair-wise modified independence LOD score as distance metric.

The independence test *G* statistic is given by:

$$ G = \sqrt {2\sum \left[ {o{\text{*ln}}\left( \frac{o}{e} \right)} \right]} $$

$o{ }$ is observed number of each genotype; $e$ is expected number in each cell.

The modified LOD score from an approximate transformation is given by:

$$ {\text{mLOD}} = \frac{{\left[ {\left( {4 - {\text{e}}^{{\frac{{ - {\text{G}}^{2} }}{{2\left( {{\text{d}} - 1} \right)}}}} } \right){\text{e}}^{{\frac{{ - {\text{G}}^{2} }}{{2\left( {{\text{d}} - 1} \right)}}}} - 3} \right]\left( {{\text{d}} - 1} \right) + {\text{G }}^{2} }}{2ln10}, $$

where $${\text{d}}$$ is the degrees of freedom; $$e$$ is the expected number, where $$ e$$ is total row multiplied by total column division by total grand.

According to Liu et al.^13^, the mapping algorithm applies an iterative process of marker ordering and error genotype correction to ensure the accuracy of map order and map distances in the presence of missing observations and genotyping errors.

### Genotyping and identification of crossover events

Based on the nucleotide at the marker sites in 950527 and Huayu22, all the candidate markers were converted to heterozygous genotypes (950527 and Huayu22) (Fig. 5a) in each individual of F_6_ and natural population (Fig. 5b). The markers on genetic map could be candidate recombination crossover events in RIL population. The recombination crossover events could be detected by the detailed methods in Yang et al.^15^. The spans with both sides of the breakpoints of ≥ 10 kb were assumed to be the outcomes of recombination crossover events^15,16^. Based on the markers’ genotypes, some regions along chromosome pairs were also converted into blocks of heterozygous genotypes or parental genotypes (Fig. 5). Slide windows (100 kb) were used for analysis of the loci recombination crossover events. Based on the heat maps and haplotype maps of each F_6_ population individual, small recombination regions (block length ≤ 200 kb) were checked to exclude potential false positives. Moreover, the recombination crossover events with ambiguous allelic relationships were excluded. The alignment gaps caused by blocks with an abnormal insert size of the paired-end reads were excluded^16^.

### Identification of gene function within hot spot regions

To identify hot spot regions of crossover events, we used a Poisson distribution to find the threshold value of the number of crossover events in each 100-kb region in 49 peanut accessions. To identify the high recombination rate regions in the peanut genome, we divided the whole peanut genome into 2920 non-overlapping windows (1000 kb for each window) and calculated the recombination rate of each window in 49 peanut accessions^16^. If the rate was ≥ 25 cMMb^−1^ (threefold of average recombination rate), we assigned this as a high recombination rate window and collected the genes in this window. A recombination rate > 50 cM per Mb indicated recombination hot spots in the RIL population (F_6_). The recombination hot spots were defined by three principles, which were less SLAF markers haplotypes region, recombination rate > 50 cM per Mb in F_6_ population, weak relative recombination of each two markers region and high recombination rate in 49 peanut accessions (> triple of genome average recombination). The gene function frequency of all the collected genes and searched for the high-frequency function compared with the whole genome in hot spot region were checked by Gene Ontology data (downloaded from the RGAP website)^16^.

## Supplementary information

Supplementary Figures.

Supplementary Table.
