# Supplementary material for: Genome-wide identification of meiotic recombination hot spots detected by SLAF in peanut (Arachis hypogaea L.)
Source: Sci Rep. 2020 Aug 14;10:13792. doi: 10.1038/s41598-020-70354-x (PMC7429841; doi:10.1038/s41598-020-70354-x)
Supplement: Supplementary file 1 — Supplementary Figures. [file 41598_2020_70354_MOESM1_ESM.pdf]

Genome-wide identification of meiotic recombination hot spots detected by SLAF in peanut (*Arachis hypogaea* L.)

XiaohuaWang[1*], Ping Xu[1*†], Yan Ren[2*], Liang Yin[2], Shuangling Li[2], Yan Wang[1], Yanmao Shi[2], Hui Li[1], Xue Cao[1], Xiaoyuan Chi[2], Tianyi Yu[2], Manish K Pandey[3], Rajeev K Varshney[3], Mei Yuan[2†]

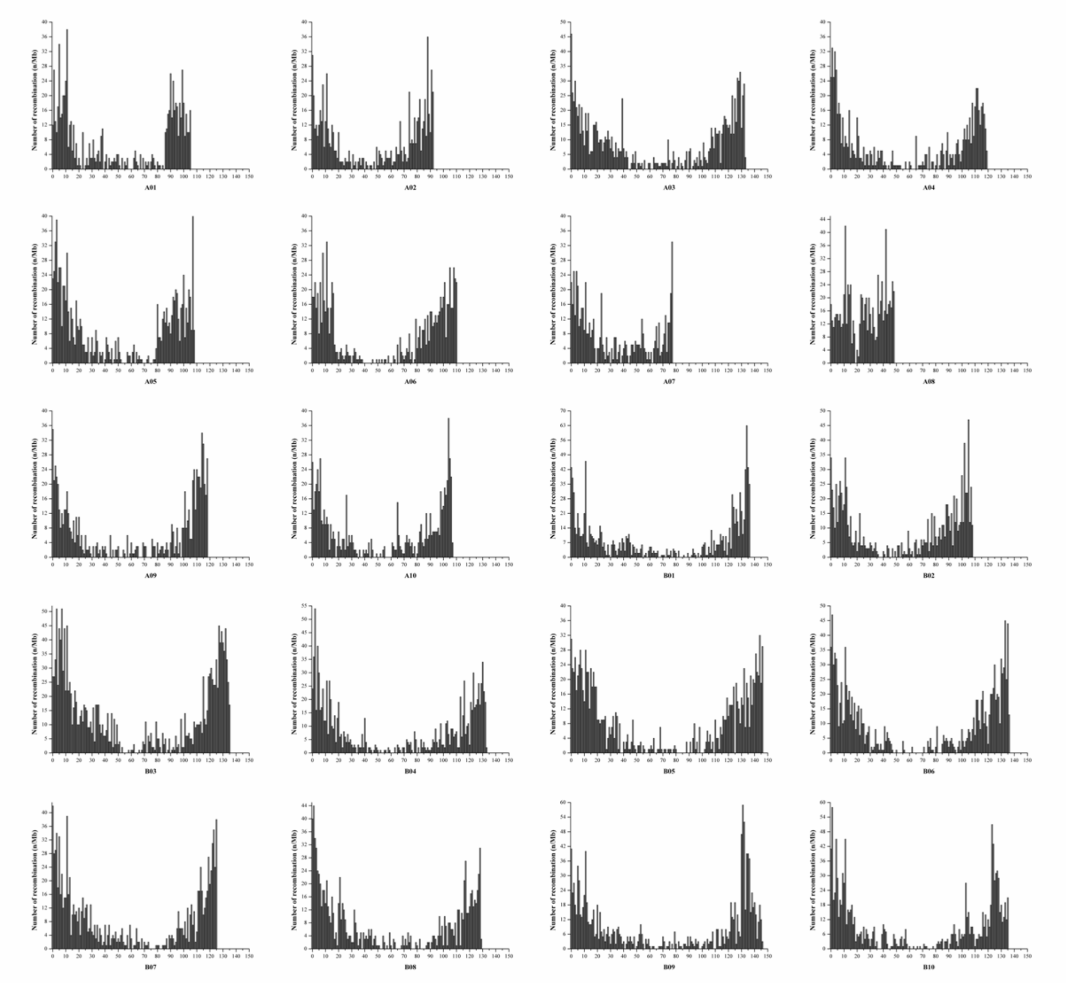

Figure S1. The recombination rates and hot spots of each chromosome in 49 peanut accessions.

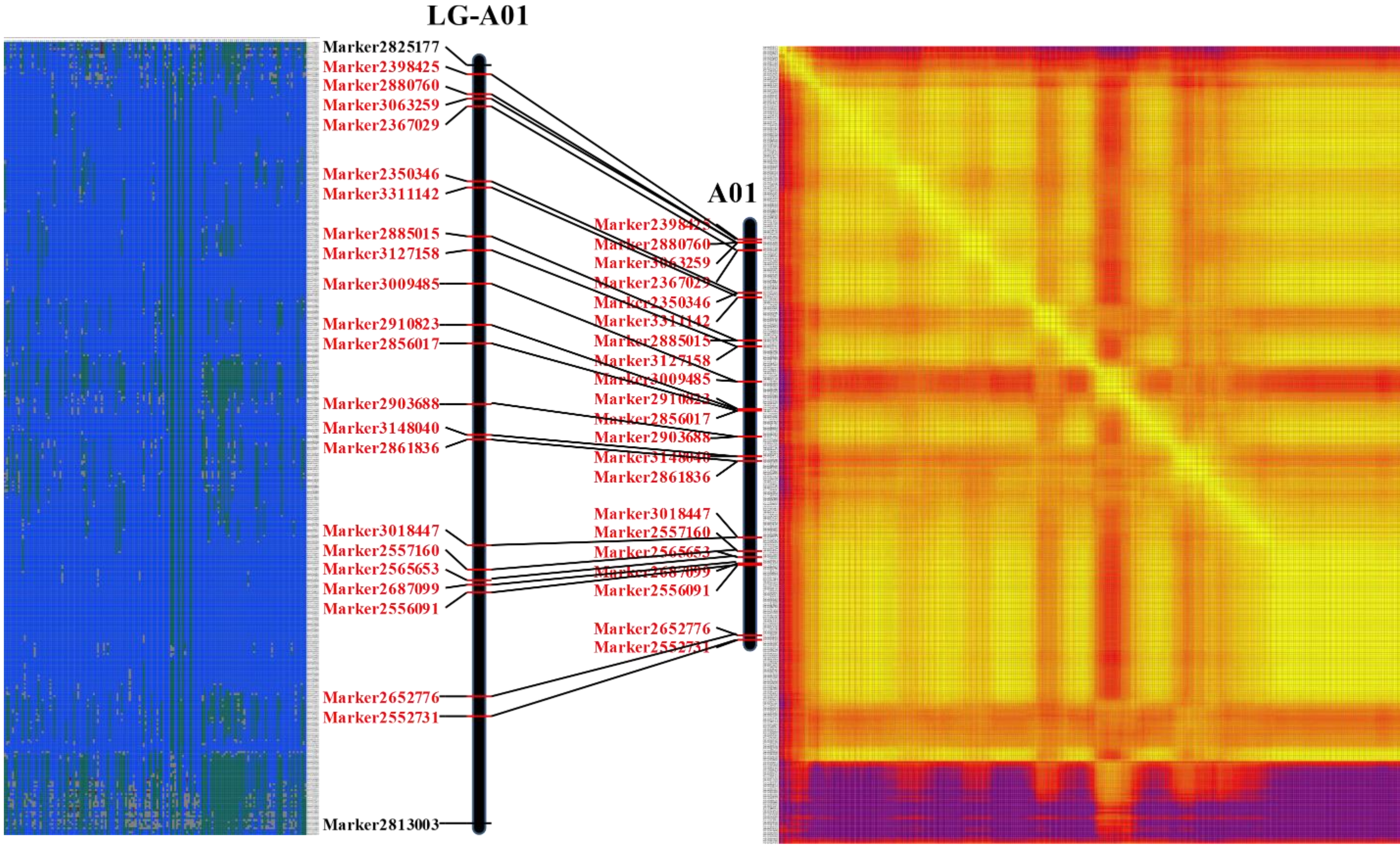

LG-A01
Marker2825177
Marker2398425
Marker2880760
Marker3063259
Marker2367029
Marker2350346
Marker3311142
Marker2885015
Marker3127158
Marker3009485
Marker2910823
Marker2856017
Marker2903688
Marker3148040
Marker2861836
Marker3018447
Marker2557160
Marker2565653
Marker2687099
Marker2556091
Marker2652776
Marker2552731
Marker2813003
A01

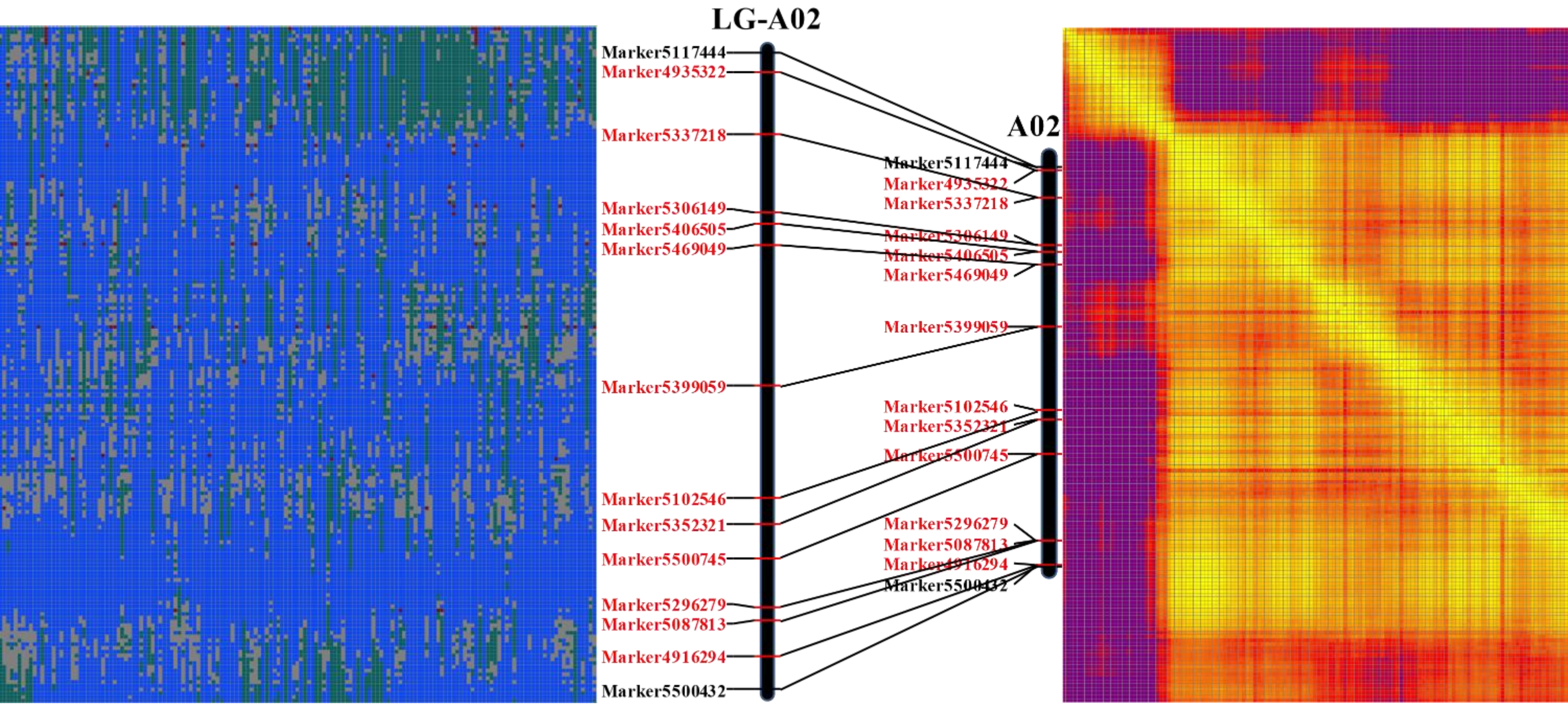
LG-A02
Marker5117444
Marker4935322
Marker5337218
Marker5306149
Marker5406505
Marker5469049
Marker5399059
Marker5102546
Marker5352321
Marker5500745
Marker5296279
Marker5087813
Marker4916294
Marker5500432
A02

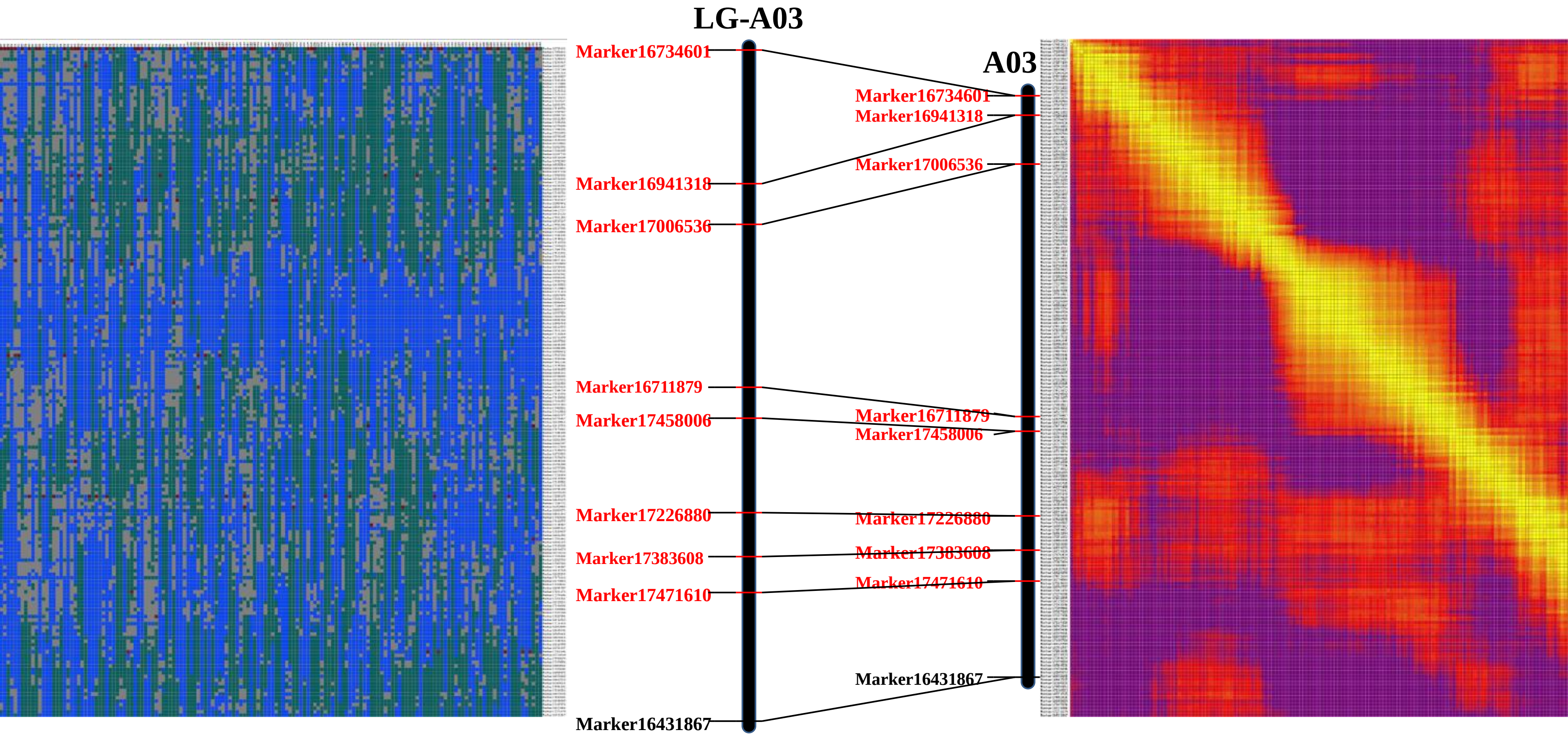

LG-A03
A03
Marker16734601
Marker16941318
Marker17006536
Marker16711879
Marker17458006
Marker17226880
Marker17383608
Marker17471610
Marker16431867
Marker16734601
Marker16941318
Marker17006536
Marker16711879
Marker17458006
Marker17226880
Marker17383608
Marker17471610
Marker16431867

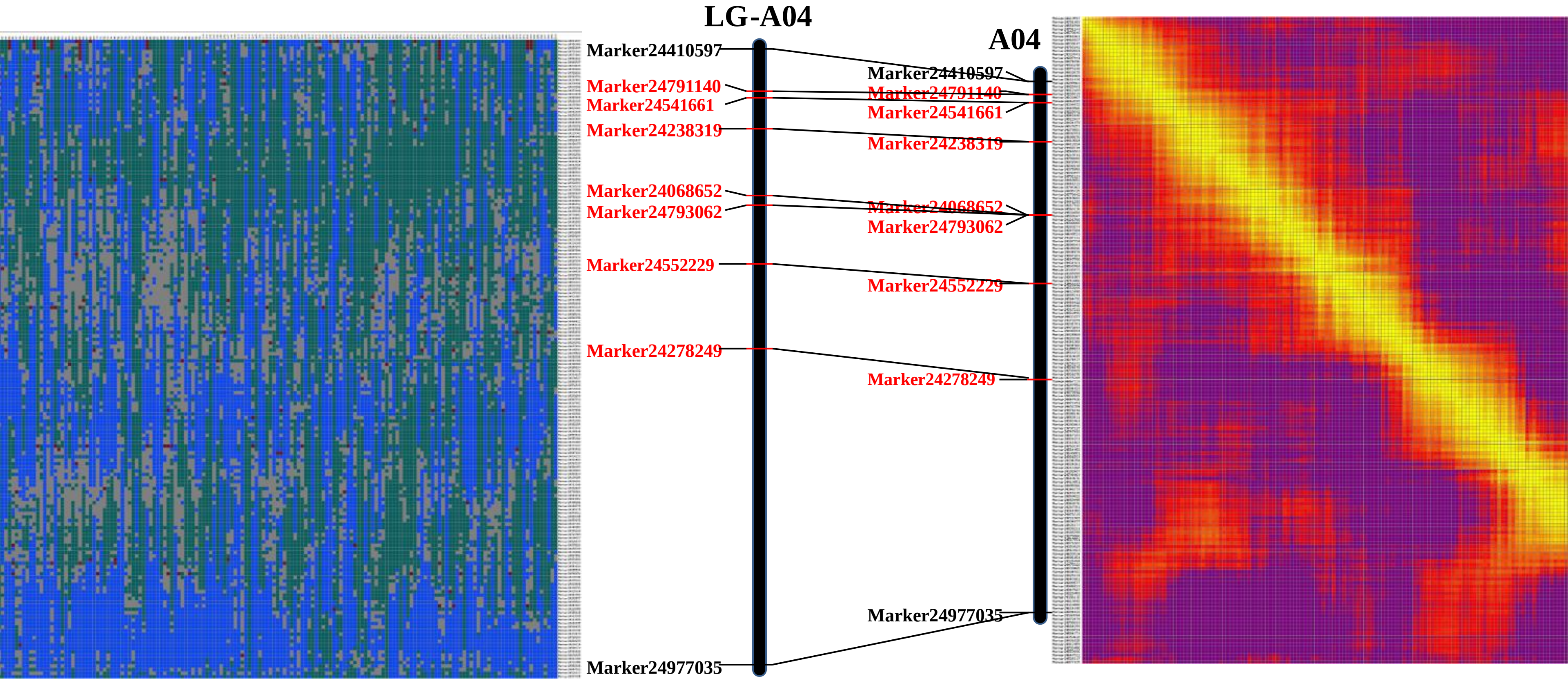

LG-A04
A04
Marker24410597
Marker24791140
Marker24541661
Marker24238319
Marker24068652
Marker24793062
Marker24552229
Marker24278249
Marker24977035
Marker24410597
Marker24791140
Marker24541661
Marker24238319
Marker24068652
Marker24793062
Marker24552229
Marker24278249
Marker24977035

LG-A05

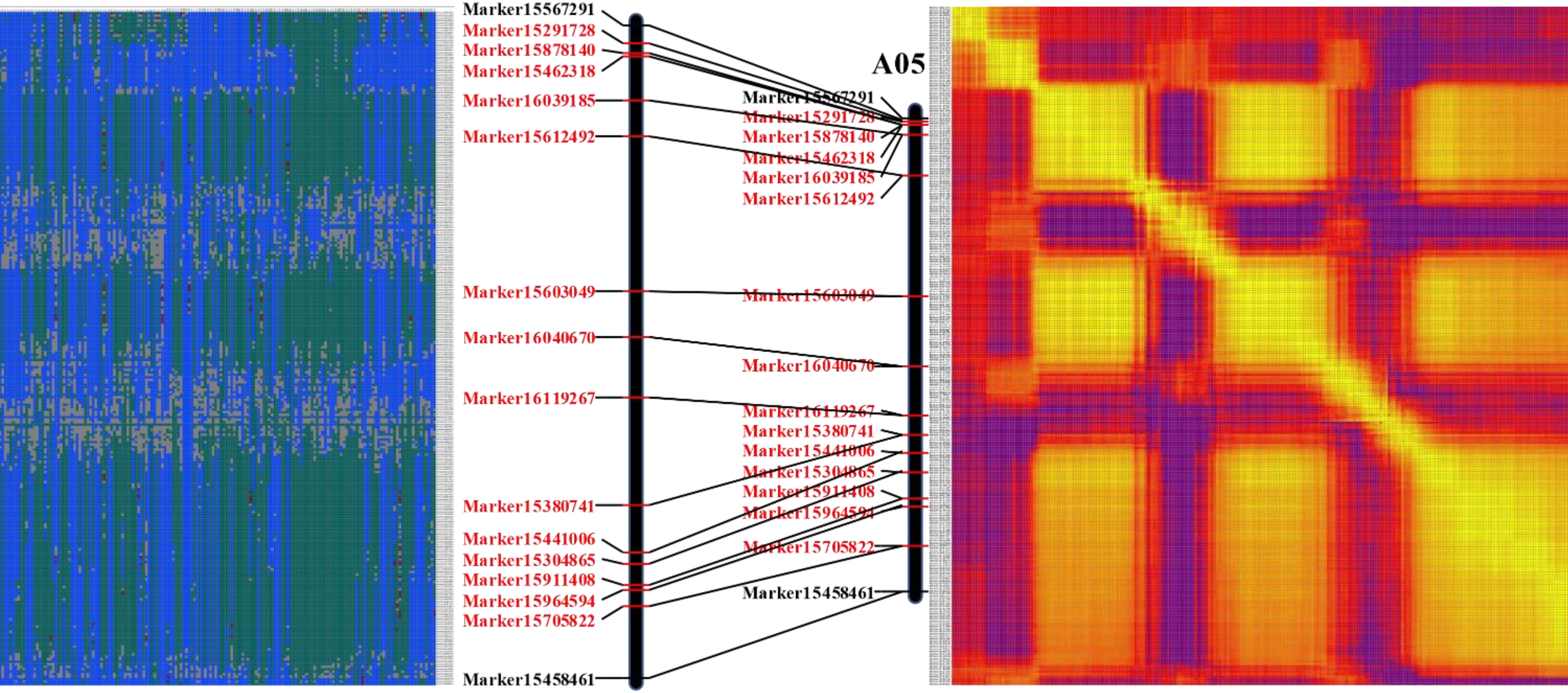
Marker15567291
Marker15291728
Marker15878140
Marker15462318
Marker16039185
Marker15612492
Marker15603049
Marker16040670
Marker16119267
Marker15380741
Marker15441006
Marker15304865
Marker15911408
Marker15964594
Marker15705822
Marker15458461
A05
Marker15567291
Marker15291728
Marker15878140
Marker15462318
Marker16039185
Marker15612492
Marker15603049
Marker16040670
Marker16119267
Marker15380741
Marker15441006
Marker15304865
Marker15911408
Marker15964594
Marker15705822
Marker15458461

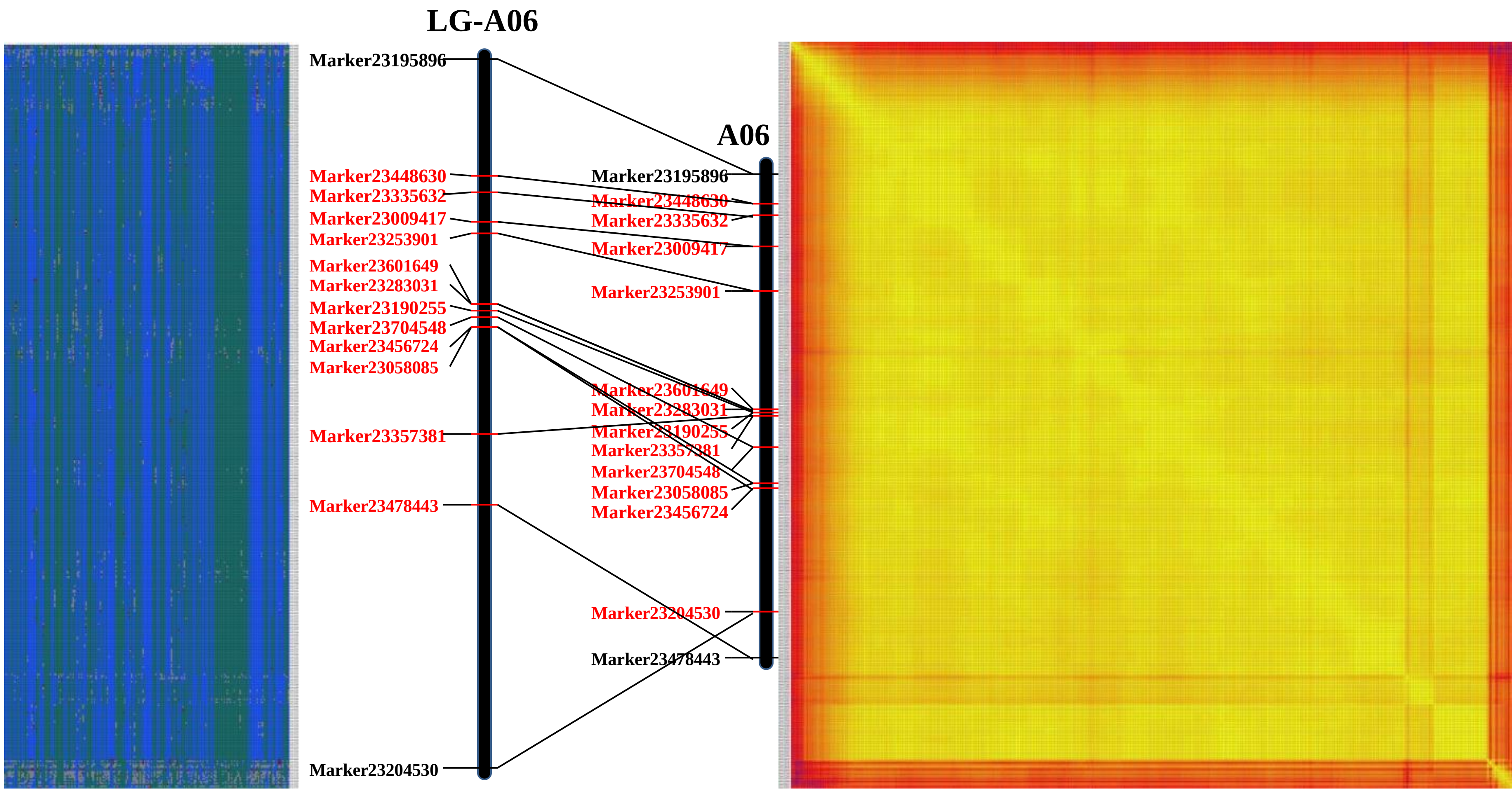

LG-A06
A06
Marker23195896
Marker23448630
Marker23335632
Marker23009417
Marker23253901
Marker23601649
Marker23283031
Marker23190255
Marker23704548
Marker23456724
Marker23058085
Marker23357381
Marker23478443
Marker23204530

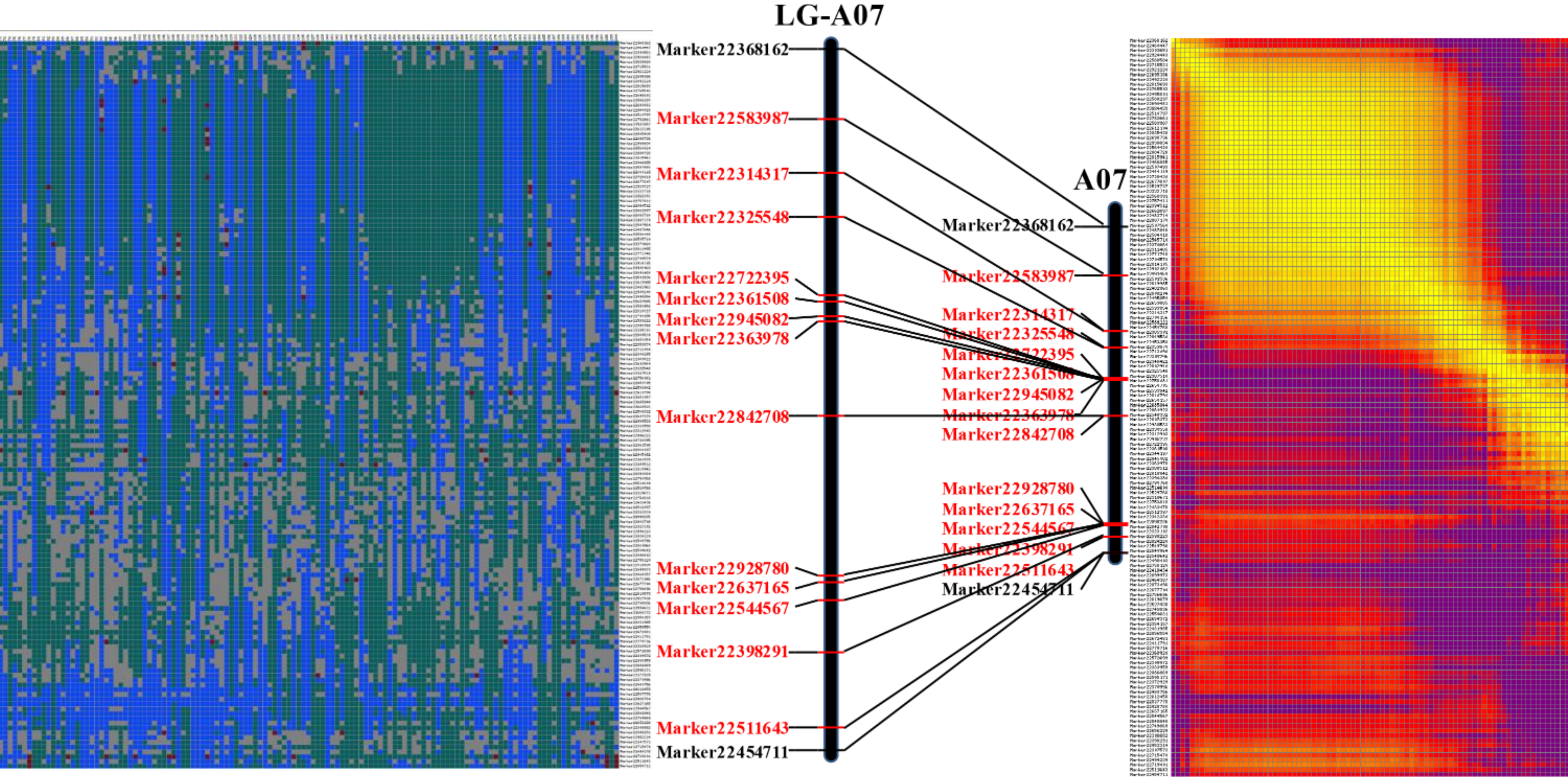

LG-A07
Marker22368162
Marker22583987
Marker22314317
Marker22325548
Marker22722395
Marker22361508
Marker22945082
Marker22363978
Marker22842708
Marker22928780
Marker22637165
Marker22544567
Marker22398291
Marker22511643
Marker22454711
A07

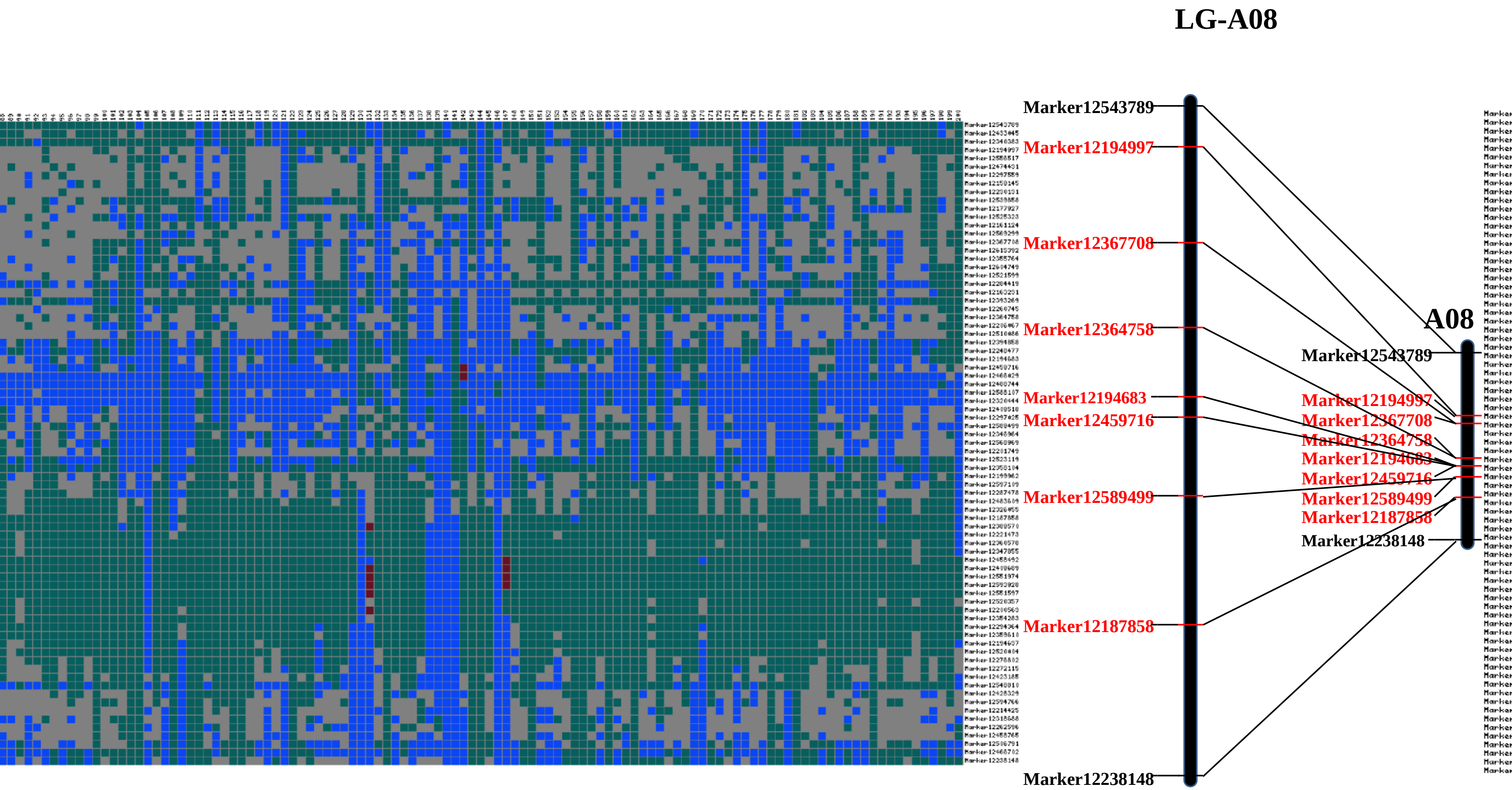
LG-A08
Marker12543789
Marker12194997
Marker12367708
Marker12364758
Marker12194683
Marker12459716
Marker12589499
Marker12187858
Marker12238148
A08
Marker12543789
Marker12194997
Marker12367708
Marker12364758
Marker12194683
Marker12459716
Marker12589499
Marker12187858
Marker12238148

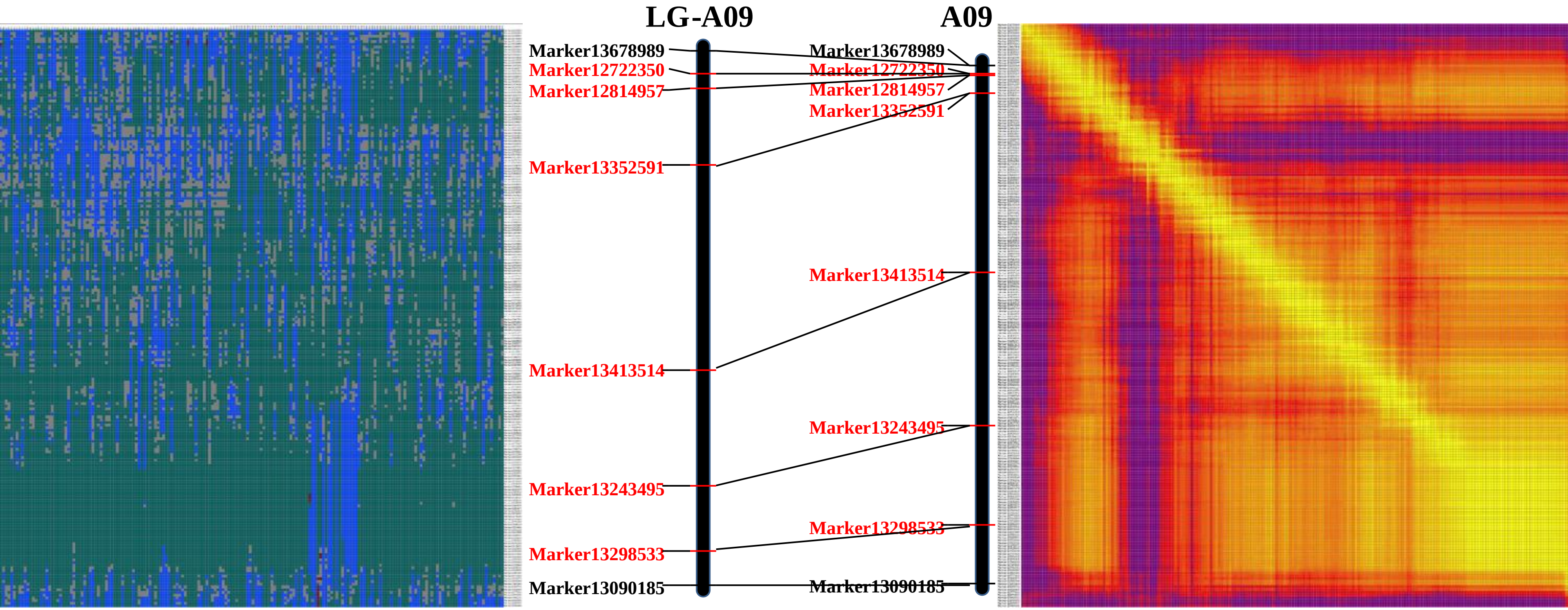
LG-A09
A09
Marker13678989
Marker12722350
Marker12814957
Marker13352591
Marker13413514
Marker13243495
Marker13298533
Marker13090185
Marker13678989
Marker12722350
Marker12814957
Marker13352591
Marker13413514
Marker13243495
Marker13298533
Marker13090185

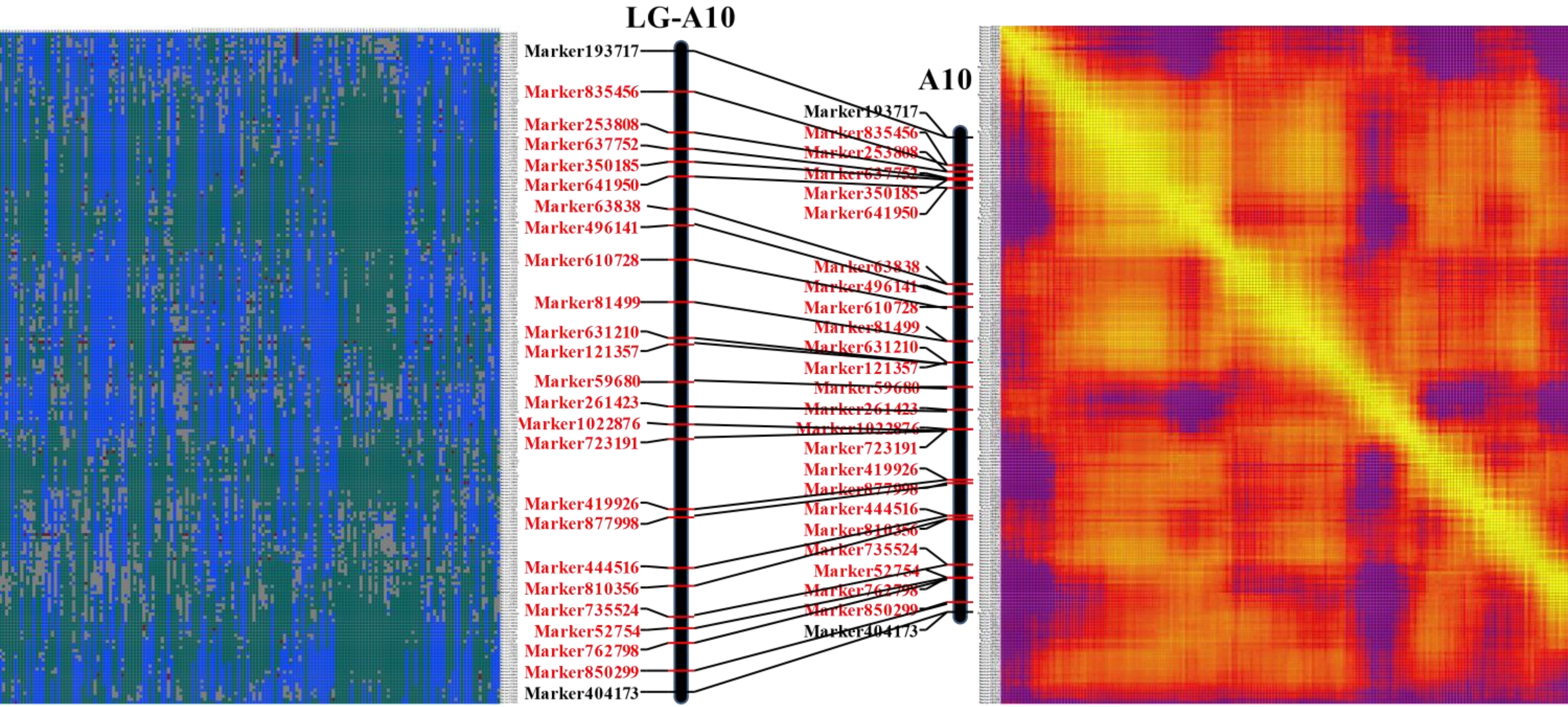
LG-A10
Marker193717
Marker835456
Marker253808
Marker637752
Marker350185
Marker641950
Marker63838
Marker496141
Marker610728
Marker81499
Marker631210
Marker121357
Marker59680
Marker261423
Marker1022876
Marker723191
Marker419926
Marker877998
Marker444516
Marker810356
Marker735524
Marker52754
Marker762798
Marker850299
Marker404173
A10
Marker193717
Marker835456
Marker253808
Marker637752
Marker350185
Marker641950
Marker63838
Marker496141
Marker610728
Marker81499
Marker631210
Marker121357
Marker59680
Marker261423
Marker1022876
Marker723191
Marker419926
Marker877998
Marker444516
Marker810356
Marker735524
Marker52754
Marker762798
Marker850299
Marker404173

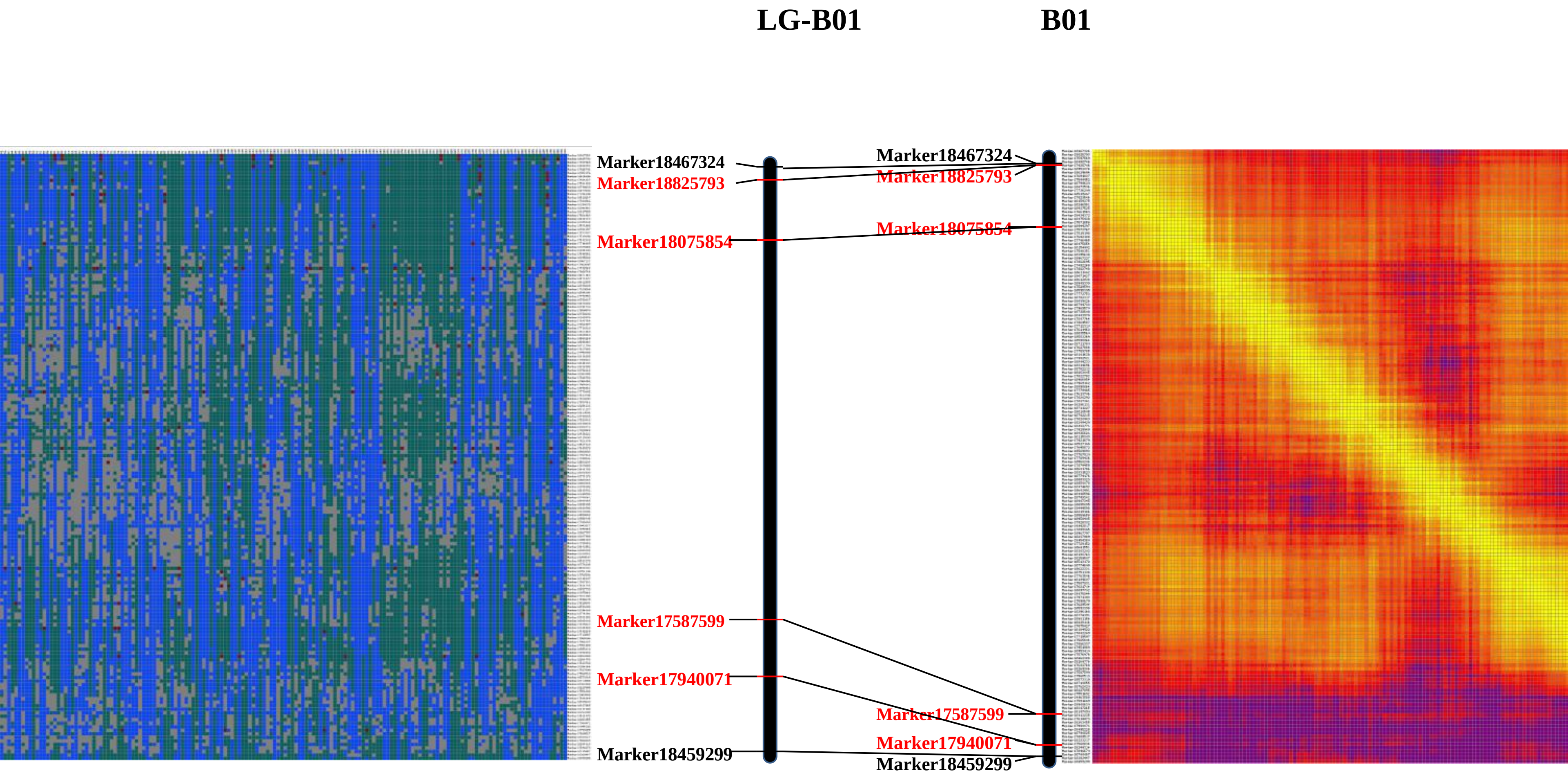
LG-B01
B01
Marker18467324
Marker18825793
Marker18075854
Marker17587599
Marker17940071
Marker18459299
Marker18467324
Marker18825793
Marker18075854
Marker17587599
Marker17940071
Marker18459299

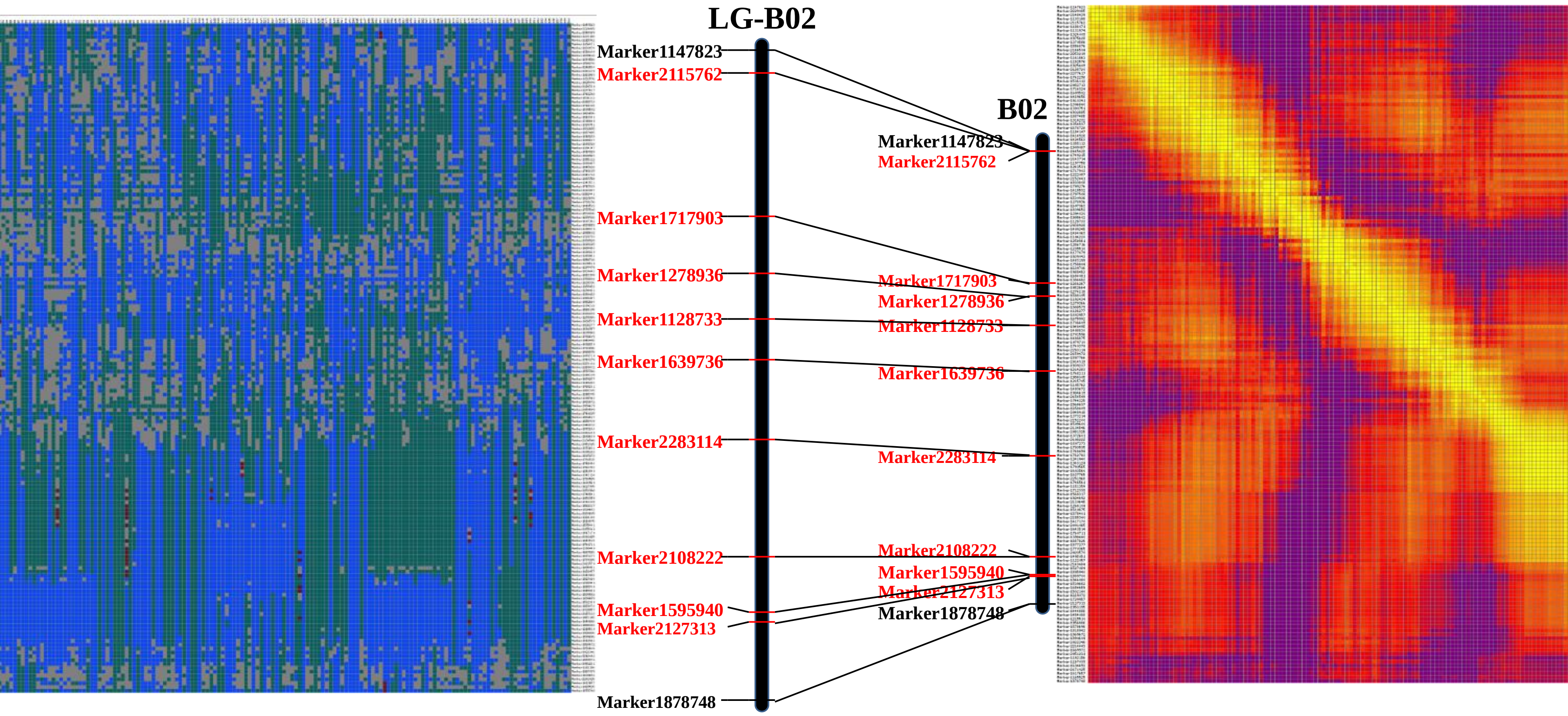

LG-B02
Marker1147823
Marker2115762
Marker1717903
Marker1278936
Marker1128733
Marker1639736
Marker2283114
Marker2108222
Marker1595940
Marker2127313
Marker1878748
B02
Marker1147823
Marker2115762
Marker1717903
Marker1278936
Marker1128733
Marker1639736
Marker2283114
Marker2108222
Marker1595940
Marker2127313
Marker1878748

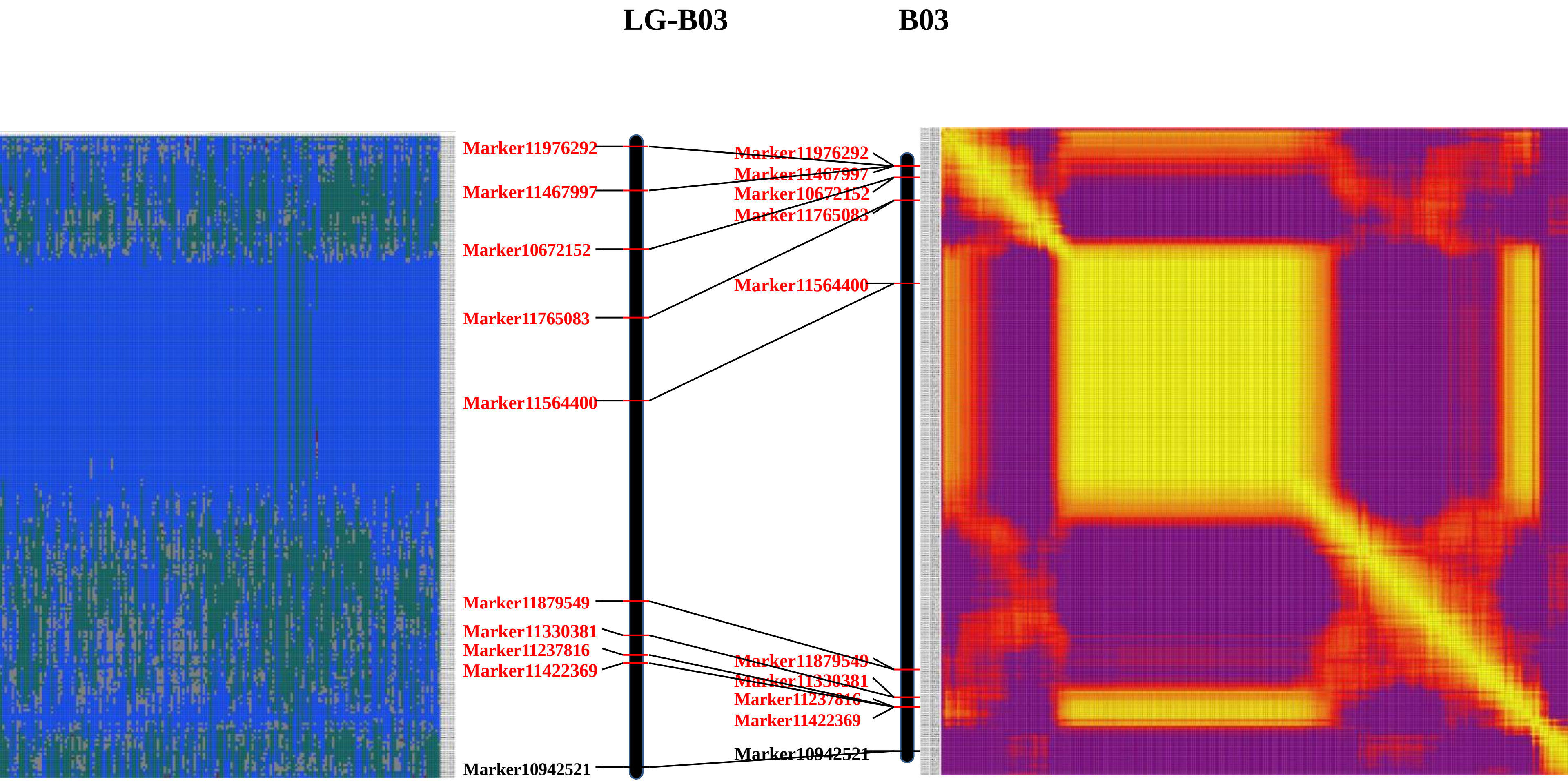

LG-B03
B03
Marker11976292
Marker11467997
Marker10672152
Marker11765083
Marker11564400
Marker11879549
Marker11330381
Marker11237816
Marker11422369
Marker10942521

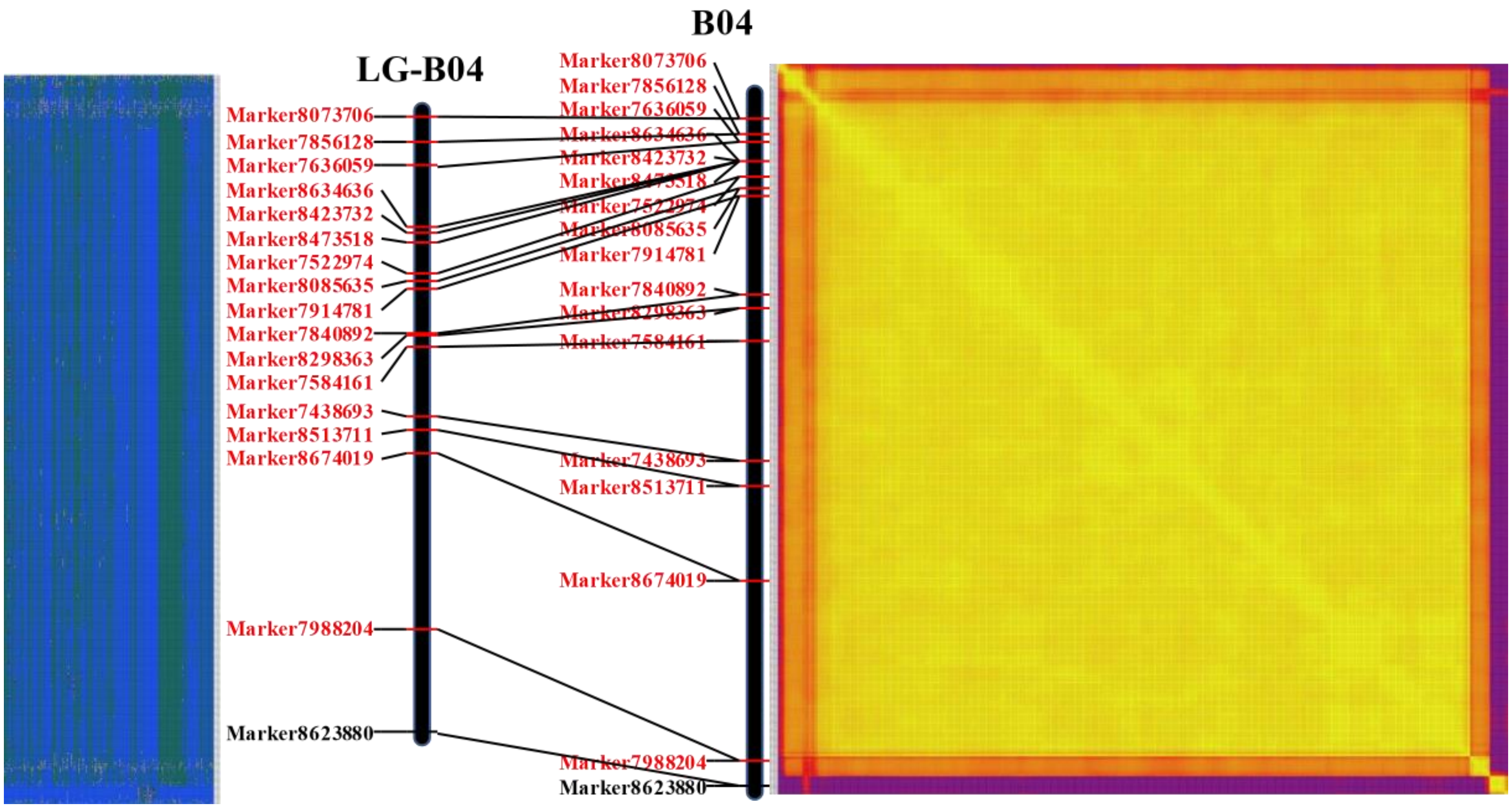
LG-B04
B04
Marker8073706
Marker7856128
Marker7636059
Marker8634636
Marker8423732
Marker8473518
Marker7522974
Marker8085635
Marker7914781
Marker7840892
Marker8298363
Marker7584161
Marker7438693
Marker8513711
Marker8674019
Marker7988204
Marker8623880

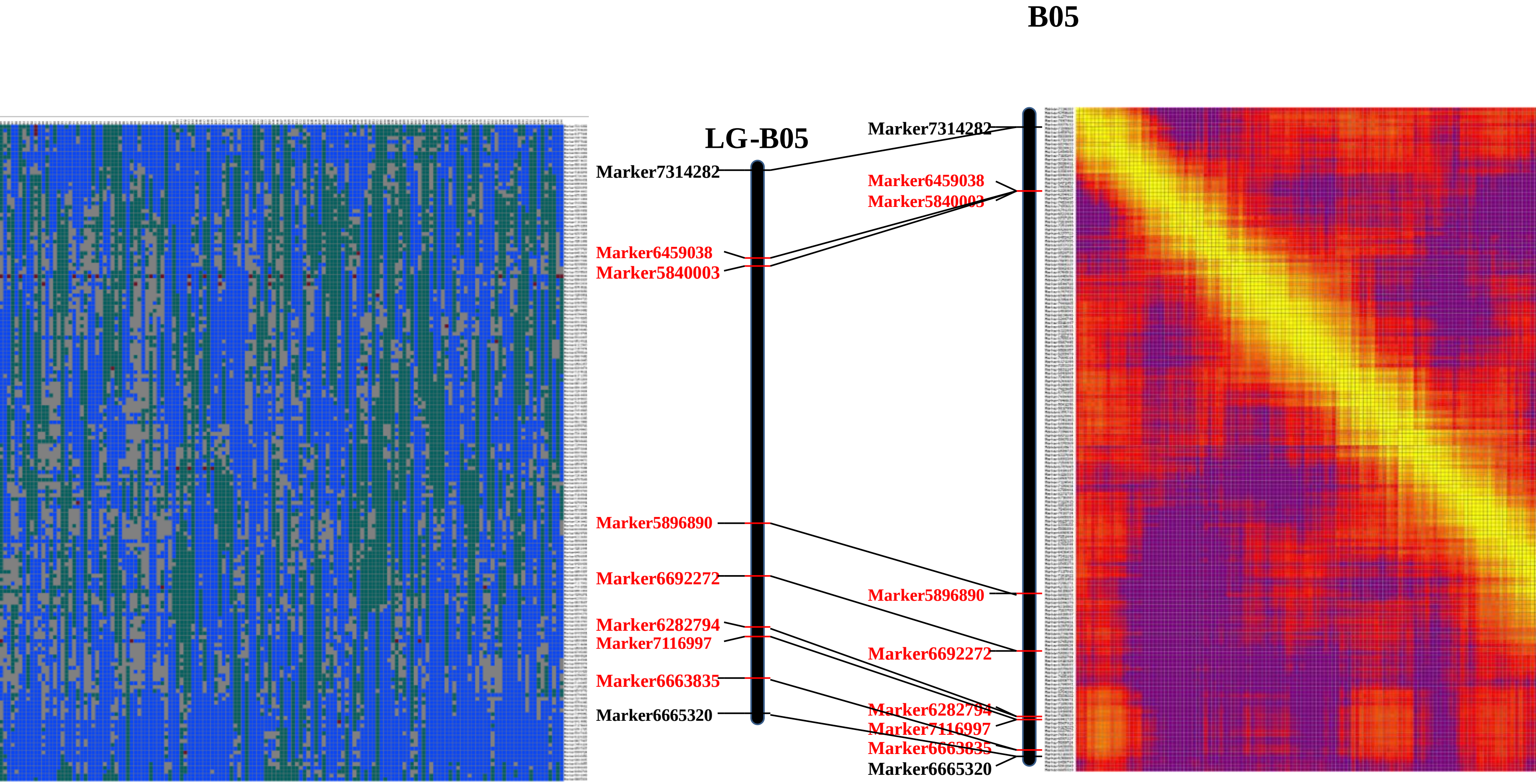
B05
LG-B05
Marker7314282
Marker6459038
Marker5840003
Marker5896890
Marker6692272
Marker6282794
Marker7116997
Marker6663835
Marker6665320
Marker7314282
Marker6459038
Marker5840003
Marker5896890
Marker6692272
Marker6282794
Marker7116997
Marker6663835
Marker6665320

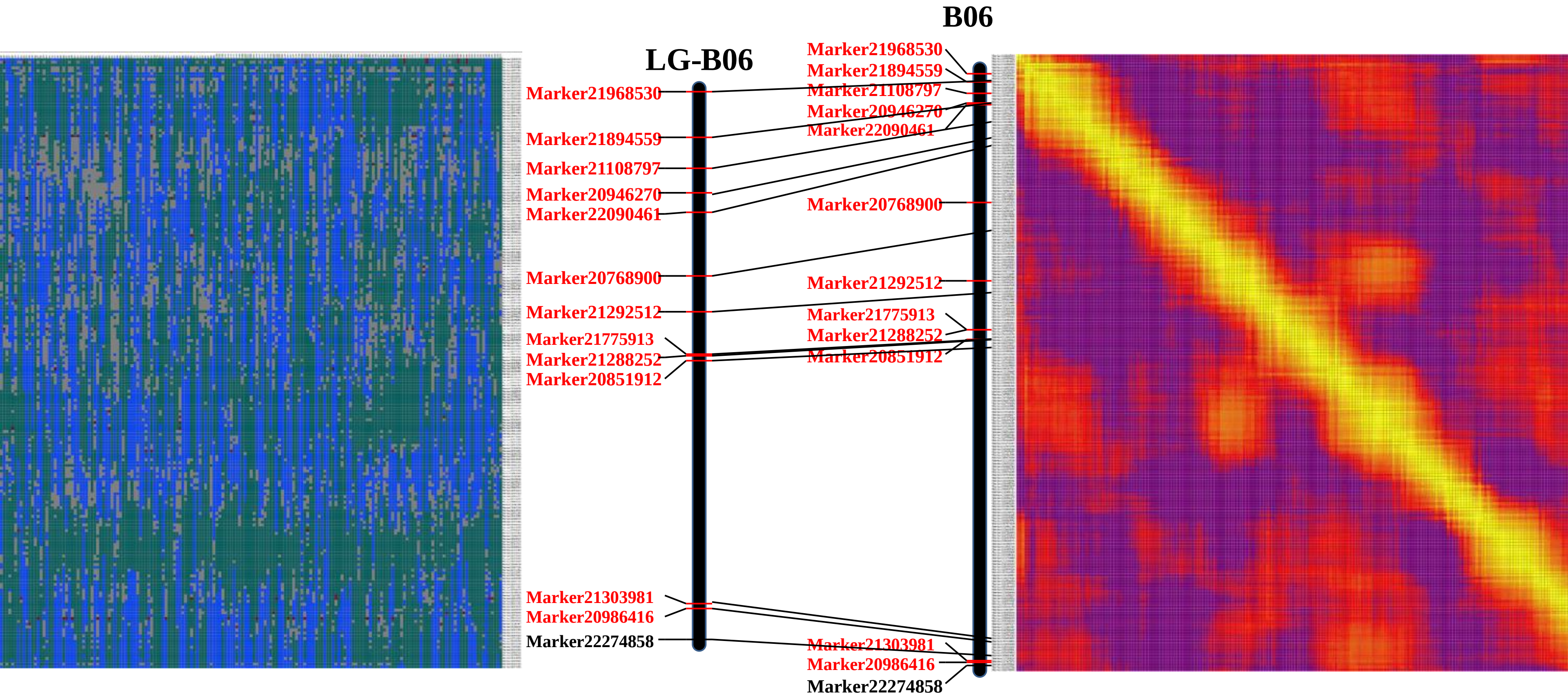

B06
LG-B06
Marker21968530
Marker21894559
Marker21108797
Marker20946270
Marker22090461
Marker20768900
Marker21292512
Marker21775913
Marker21288252
Marker20851912
Marker21303981
Marker20986416
Marker22274858
Marker21968530
Marker21894559
Marker21108797
Marker20946270
Marker22090461
Marker20768900
Marker21292512
Marker21775913
Marker21288252
Marker20851912
Marker21303981
Marker20986416
Marker22274858

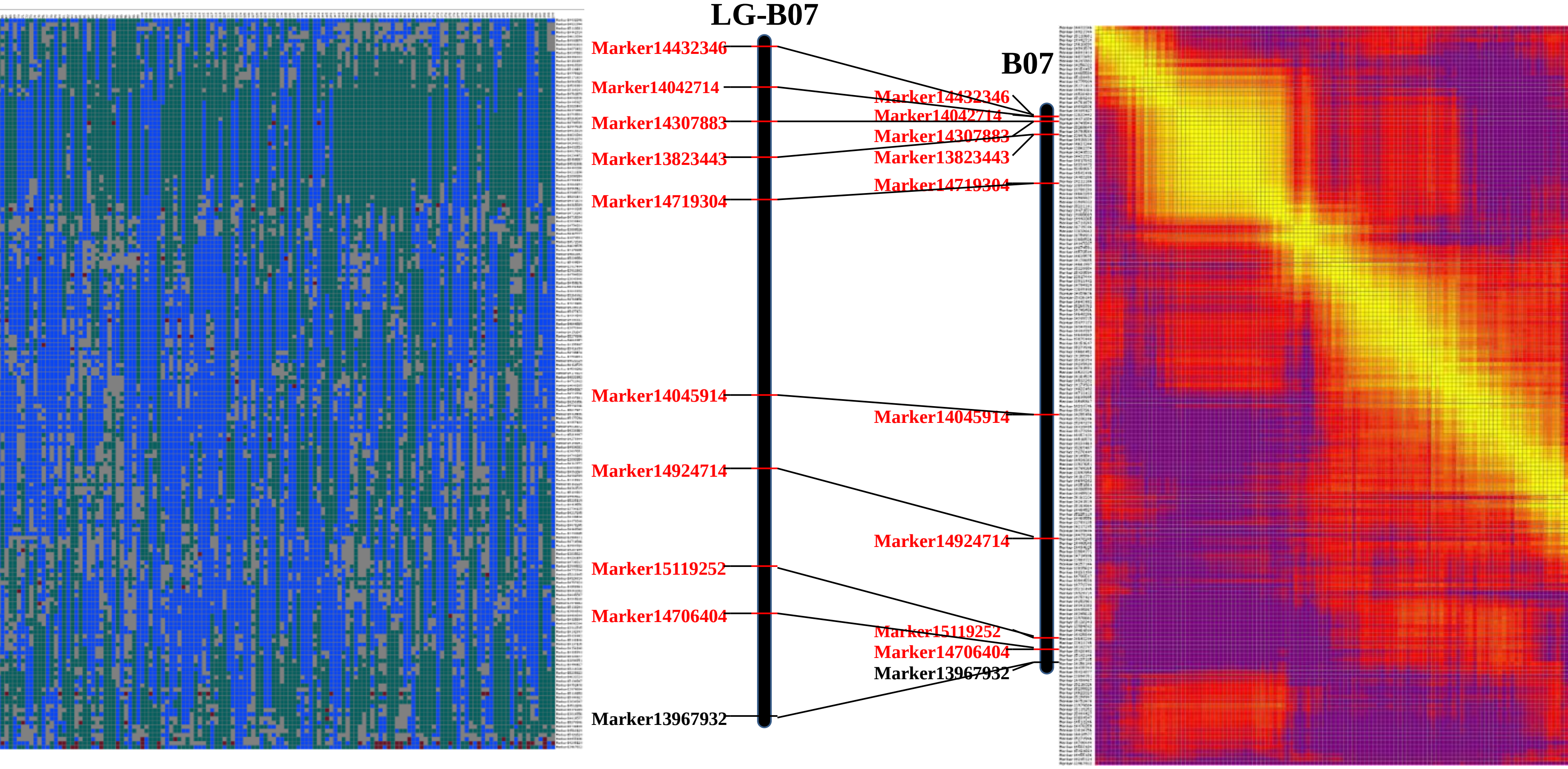
LG-B07
Marker14432346
Marker14042714
Marker14307883
Marker13823443
Marker14719304
Marker14045914
Marker14924714
Marker15119252
Marker14706404
Marker13967932
B07
Marker14432346
Marker14042714
Marker14307883
Marker13823443
Marker14719304
Marker14045914
Marker14924714
Marker15119252
Marker14706404
Marker13967932

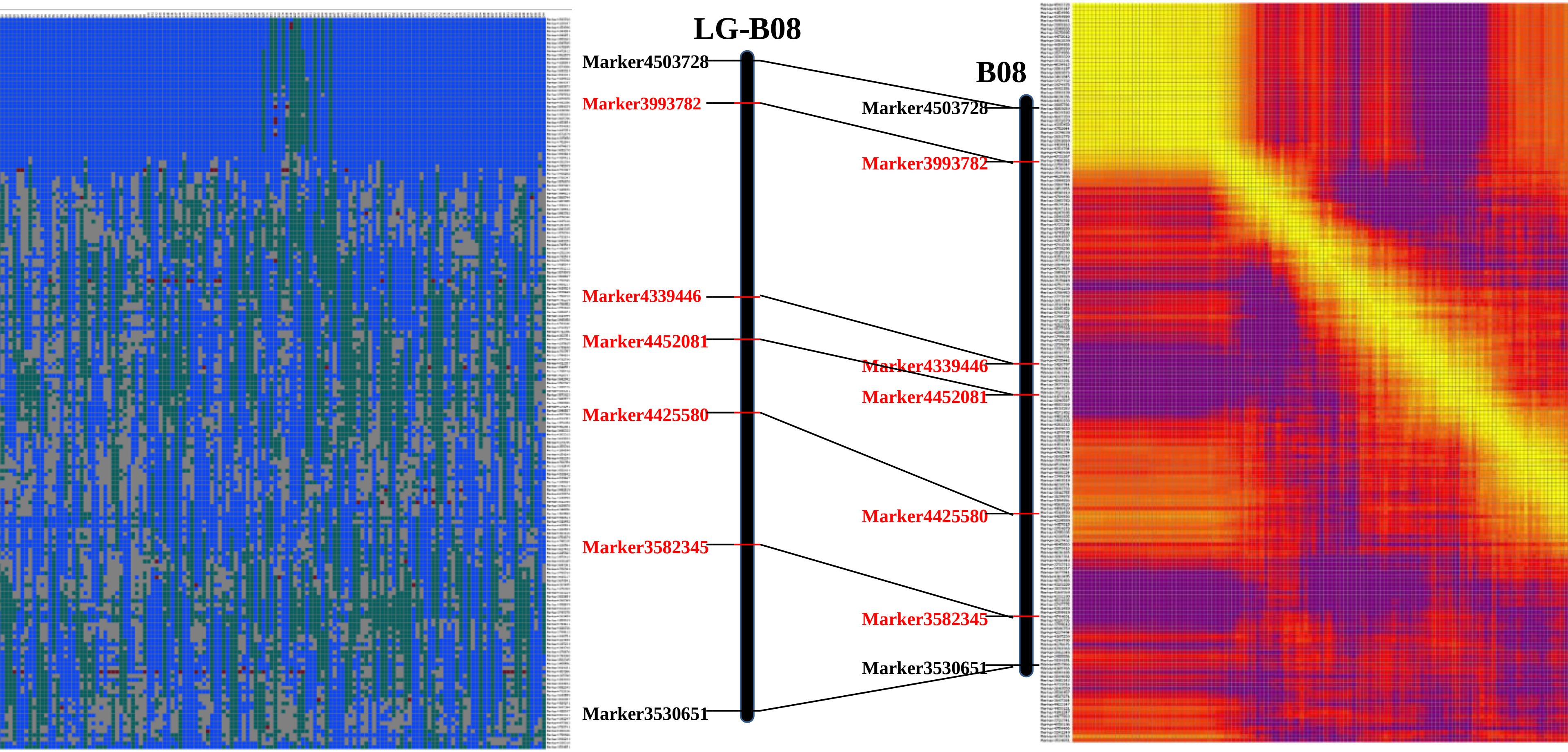
LG-B08
Marker4503728
Marker3993782
Marker4339446
Marker4452081
Marker4425580
Marker3582345
Marker3530651
B08
Marker4503728
Marker3993782
Marker4339446
Marker4452081
Marker4425580
Marker3582345
Marker3530651

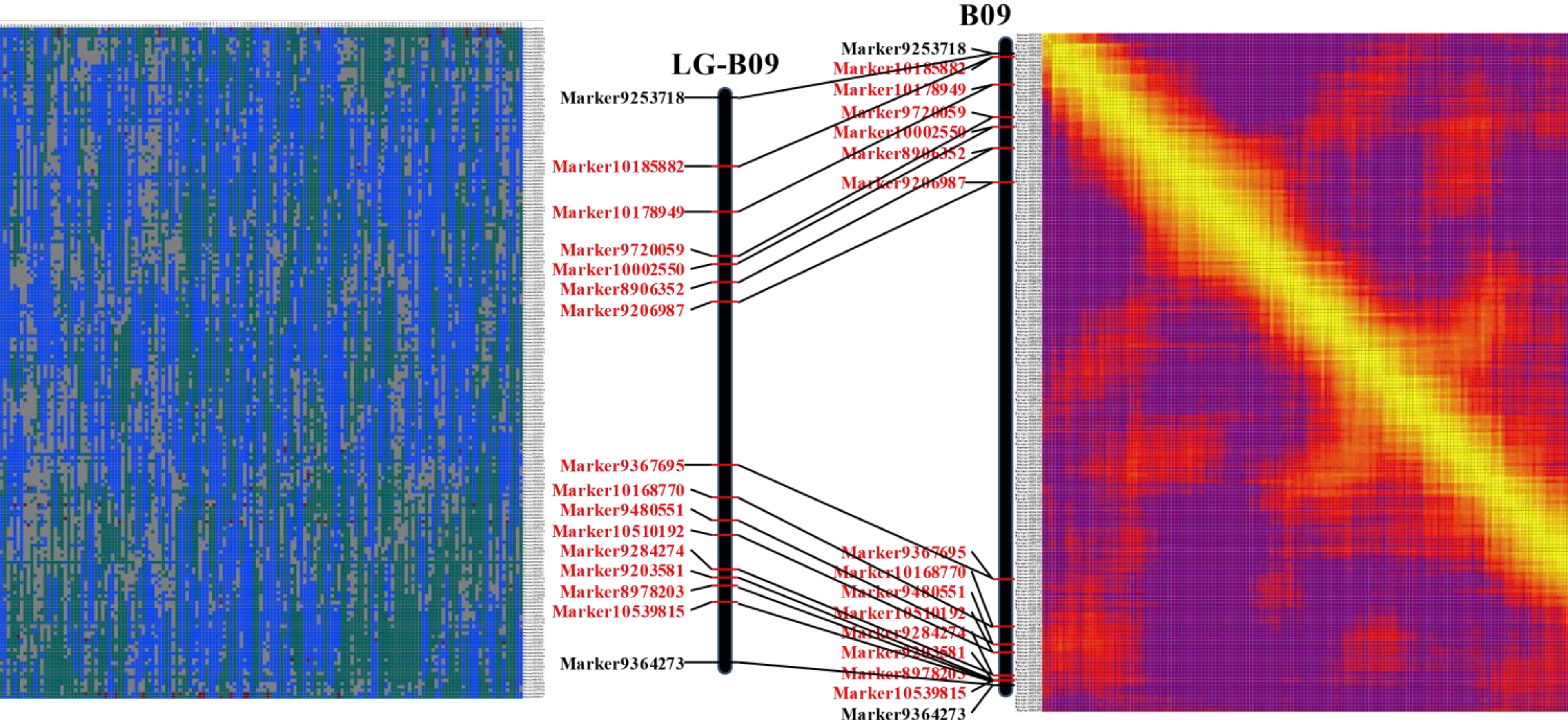
LG-B09
Marker9253718
Marker10185882
Marker10178949
Marker9720059
Marker10002550
Marker8906352
Marker9206987
Marker9367695
Marker10168770
Marker9480551
Marker10510192
Marker9284274
Marker9203581
Marker8978203
Marker10539815
Marker9364273
B09
Marker9253718
Marker10185882
Marker10178949
Marker9720059
Marker10002550
Marker8906352
Marker9206987
Marker9367695
Marker10168770
Marker9480551
Marker10510192
Marker9284274
Marker9203581
Marker8978203
Marker10539815
Marker9364273

LG-B10 B10

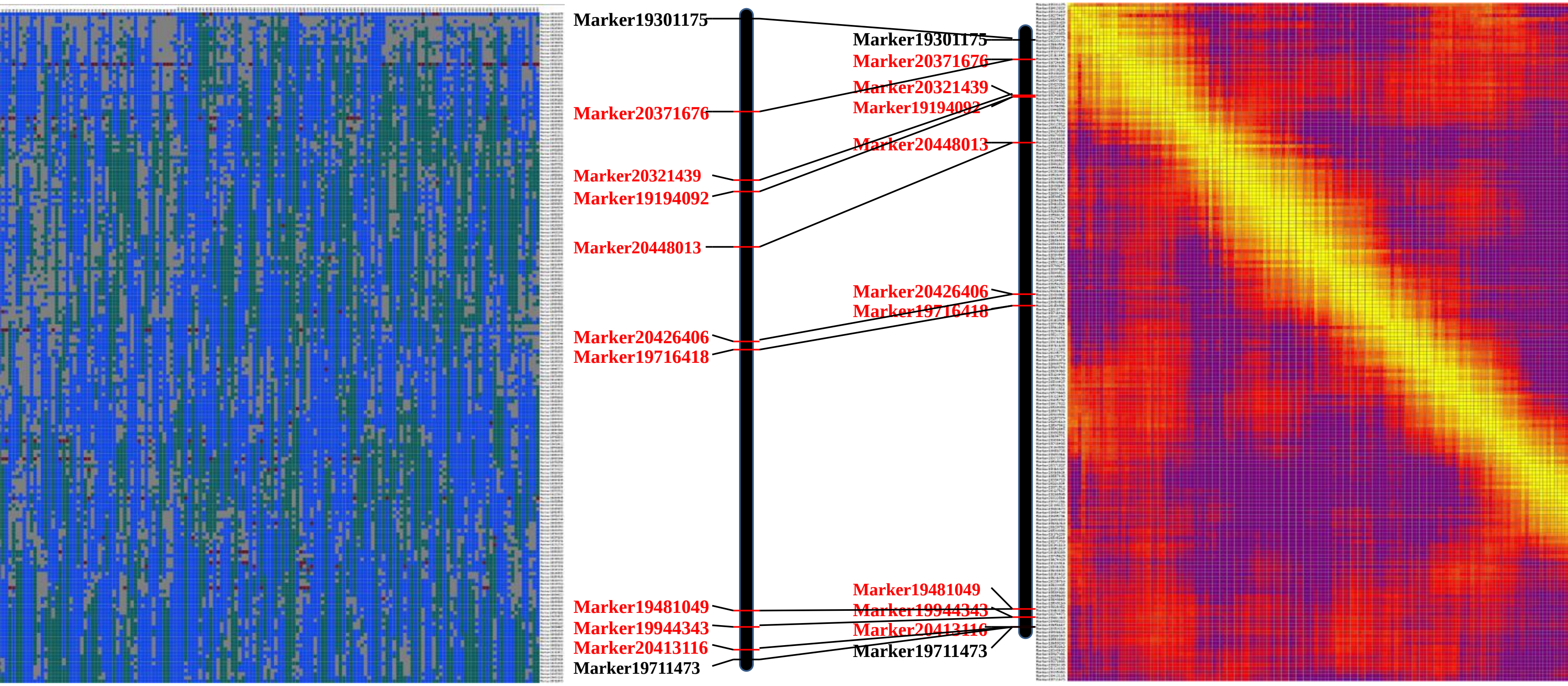
Marker19301175
Marker20371676
Marker20321439
Marker19194092
Marker20448013
Marker20426406
Marker19716418
Marker19481049
Marker19944343
Marker20413116
Marker19711473
Marker19301175
Marker20371676
Marker20321439
Marker19194092
Marker20448013
Marker20426406
Marker19716418
Marker19481049
Marker19944343
Marker20413116
Marker19711473
